# Concordance of recommendations across clinical practice guidelines for the management of hypertension in Southeast Asia with internationally reputable sources

**DOI:** 10.1186/s12872-021-02054-x

**Published:** 2021-07-28

**Authors:** Chia Siang Kow, Syed Shahzad Hasan, Pei Se Wong, Rohit Kumar Verma

**Affiliations:** 1 School of Pharmacy, Monash University Malaysia, Bandar Sunway, Selangor Malaysia; 2 Department of Pharmacy, University of Huddersfield, Huddersfield, UK; 3 School of Biomedical Sciences and Pharmacy, University of Newcastle, Callaghan, Australia; 4 School of Pharmacy, International Medical University, Kuala Lumpur, Malaysia

## Abstract

**Objectives:**

This study aimed to assess the rate of concordance, and to investigate sources of non-concordance of recommendations in the management of hypertension across CPGs in Southeast Asia, with internationally reputable clinical practice guidelines (CPGs).

**Methods:**

CPGs for the management of hypertension in Southeast Asia were retrieved from the websites of the Ministry of Health or cardiovascular specialty societies of the individual countries of Southeast Asia during November to December 2020. The recommendations for the management of hypertension specified in the 2017 American College of Cardiology (ACC)/American Heart Association (AHA) guideline and the 2018 European Society of Cardiology (ESC)/European Society of Hypertension (ESH) guideline were selected to be the reference standards; the recommendations concerning the management of hypertension in the included CPGs in Southeast Asia were assessed if they were concordant with the reference recommendations generated from both the 2017 ACC/AHA guideline and the 2018 ESC/ESH guideline, using the population (P)-intervention (I)-comparison (C) combinations approach.

**Results:**

A total of 59 reference recommendations with unique and unambiguous P-I-C specifications was generated from the 2017 ACC/AHA guideline. In addition, a total of 51 reference recommendations with unique and unambiguous P-I-C specifications was generated from the 2018 ESC/ESH guideline. Considering the six included CPGs from Southeast Asia, concordance was observed for 30 reference recommendations (50.8%) out of 59 reference recommendations generated from the 2017 ACC/AHA guideline and for 31 reference recommendations (69.8%) out of 51 reference recommendations derived from the 2018 ESC/ESH guideline.

**Conclusions:**

Hypertension represents a significant issue that places health and economic strains in Southeast Asia and demands guideline-based care, yet CPGs in Southeast Asia have a high rate of non-concordance with internationally reputable CPGs. Concordant recommendations could perhaps be considered a standard of care for hypertension management in the Southeast Asia region.

**Supplementary Information:**

The online version contains supplementary material available at 10.1186/s12872-021-02054-x.

## Introduction

Over the past decade, Southeast Asia has undergone significant socioeconomic development, which led to changes in lifestyle that translated into a growing prevalence of chronic non-communicable diseases. Approximately one-third of adults in the region have hypertension and close to 1.5 million deaths are attributed to hypertension annually [1]. Hypertension represents a significant issue that places health and economic strains in Southeast Asia, as this is partly due in part to absent or poor disease management, with rates of uncontrolled hypertension reported in some Southeast Asian countries were as high as 80% [2–20].

Proper management of hypertension which could reduce the risk of cardiovascular morbidity and mortality, signifies the importance of clinical practice guidelines (CPGs) to guide clinicians in the accurate diagnosis and appropriate treatment of patients with hypertension [21]. By far the two most well-established CPGs for the management of hypertension are the 2017 American College of Cardiology (ACC)/American Heart Association (AHA) guideline [22] and the 2018 European Society of Cardiology (ESC)/European Society of Hypertension (ESH) guideline [23]. The 2017 ACC/AHA guideline [22] recommended tighter blood pressure control (systolic blood pressure of < 130 mmHg and diastolic blood pressure of < 80 mmHg), which is backed by several meta-analyses of observational studies which reported significantly higher hazards for the development of cardiovascular disease and stroke with blood pressure of ≥ 120–129/80–84 mm Hg relative to blood pressure of < 120/80 mm Hg (hazard ratios ranged from 1.1 to 1.5) [24]. In addition, evidence for the positive outcomes from tighter blood pressure control was also reported in the Systolic Blood Pressure Intervention (SPRINT) trial [25].

Rather than focusing on tighter blood pressure control, the 2018 ESC/ESH guideline [23] defines hypertension based on the level of blood pressure at which the benefits either with lifestyle interventions or treatment with antihypertensive agents, outweighed their risks. Indeed, such recommendation is supported by the meta-analyses of randomized controlled trials (instead of the meta-analyses of observational studies cited in the 2017 ACC/AHA guideline [22]), which demonstrated that treatment of patients with blood pressure of ≥ 140/90 mm Hg was considered beneficial [23]. Moreover, observational cohort studies with high-risk and older population, as referenced in the 2017 ACC/AHA guideline [22], could increase the detection of statistical significance over a shorter follow-up period. The discrepancies in the type of evidence used between the 2017 ACC/AHA guideline [22] and the 2018 ESC/ESH guideline [23] may provide insight as to why the recommended blood pressure thresholds for the treatment and diagnosis hypertension differ between the two well-established CPGs.

Although low- and middle-income regions including countries in the Southeast Asia have developed CPGs for the management of hypertension, they often follow closely the release of CPGs from the high-income regions, and these CPGs are adopted/adapted from those of the high-income regions in many instances [26]. Nevertheless, common in both low and high-income regions, the non-concordance in recommendations in the CPGs for the management of hypertension frequently cause confusion among health care providers [27]. Only a few studies had addressed if the recommendations in the CPGs for management of hypertension originated from the Southeast Asia are concordant with internationally reputable sources.

Al-Ansary et al. [28] compared the recommendations in 11 CPGs for the management of hypertension, one of which was the CPG originated from Southeast Asia (Malaysia). There were disagreements in terms of the recommendations for pharmacotherapy of hypertension. For instance, it was reported that the included CPGs were discrepant in the strategies for adjustment of antihypertensive agents. Most CPGs recommended adding an antihypertensive agent from another class if the blood pressure has not been well-controlled; but few CPGs recommended substituting with another antihypertensive agent, with or without increasing the dose of the initial antihypertensive agent. Moreover, recommendations related to the combination of antihypertensive agents also differed across CPGs.

It is noteworthy that concordance of recommendations across CPGs is one of the factors that could affect the implementation of the recommendations specified in CPGs in the clinical practice. Therefore, this study aimed to assess the rate of concordance of recommendations in the management of hypertension across CPGs in Southeast Asia with internationally reputable CPGs and to investigate the sources of non-concordance in recommendations in the management of hypertension across CPGs in Southeast Asia with internationally reputable CPGs.

## Methods

### Identification and selection of clinical practice guidelines

Two investigators (CSK and SSH) independently searched and identified the CPGs for the management of hypertension in Southeast Asia during November 2020 to December 2020 from the websites of the Ministry of Health or cardiovascular specialty societies of the individual countries of Southeast Asia. If the CPGs cannot be identified from the websites, the Ministry of Health or cardiovascular specialty societies of the respective country was approached formally by email to request a softcopy of the CPG. Only the latest version of CPGs was selected for inclusion if the CPGs had several versions. 

In order to ensure all potentially relevant CPGs had been identified, the two investigators (CSK and SSH) also independently conducted targeted searching of CPGs by country in Turning Research into Practice database, Google Scholar database, and Google search engine using the keywords “hypertension”, “high blood pressure”, “clinical practice guideline”, “guideline”, “recommendation”, and “consensus”. The reference lists of included CPGs were also manually examined for potentially relevant CPGs. Any discrepancies in the selection of CPGs for inclusion were resolved through consensus discussion with a third investigator.

The eligibility criteria for the selection of CPGs from each respective country in Southeast Asia included: (1) CPGs that were currently active for use by health care providers in the respective country at the time of selection; (2) CPGs that were published/endorsed by the Ministry of Health or cardiovascular specialty societies of the respective country; (3) CPGs that were developed or updated in or after 2010; (4) CPGs that were published in official (translated) language of English or Malay; (5) CPGs that were regarded as the principal source of guidance for clinical care of hypertension by health care practitioners in the respective country at the time of selection; and (6) CPGs that addressed the general management of hypertension (e.g., goals blood pressure or pharmacotherapy in patients with hypertension with or without comorbidities).

Exclusion criteria included: (1) CPGs that were published in official (translated) languages other than English or Malay; (2) CPGs that addressed the management of hypertension in patients with specific comorbidities (e.g., diabetes, stroke, or cardiovascular disease); and (3) documents with single author or publications such as summaries of CPGs and non-official translated versions of CPGs.

### Data extraction from the included clinical practice guidelines

Two investigators (CSK and SSH) independently performed data extraction from the included CPGs with a pre-designed data extraction form. Discrepancies in the extracted data were resolved by consensus, involving other investigators if necessary. The following information was extracted: the publication year, status of the CPG (newly developed or updated from the previous version), the type of elaboration organization (governmental institution or specialty society), availability of funding/sponsorship, size of CPG development group, total number of cited references, total number of cited systematic reviews, total number of cited Cochrane reviews, and evidence classification method.

### Reference recommendation specification

The 2017 ACC/AHA guideline [22] and the 2018 ESC/ESH guideline [23] for the management of hypertension represented two of the most influential CPGs in the domain of hypertension which could inform the treatment practice of hypertension globally at the time of conducting this study. In fact, both the 2017 ACC/AHA guideline [22] and the 2018 ESC/ESH guideline [23] for the management of hypertension were in the top 5% of all research outputs ever tracked by Altmetric and has been cited in more than 2,400 publications and 1,800 publications respectively, as tracked by Web of Science, as of November 2020. Therefore, the recommendations for the management of hypertension specified in the 2017 ACC/AHA guideline [22] and the 2018 ESC/ESH guideline [23] were selected to be the reference standards (aka reference recommendations); the recommendations concerning the management of hypertension in the included CPGs in Southeast Asia were assessed if they were concordant with the reference recommendations generated respectively from the 2017 ACC/AHA guideline [22] and the 2018 ESC/ESH guideline [23].

However, differences in the interpretations of population, intervention, and comparator concepts for a given reference recommendation precluded direct analysis of concordance of recommendations. In order to avoid ambiguity that might arise, generation of the reference recommendations was adapted from a previously validated population (P)-intervention (I)-comparison (C) combinations approach [29] in order to provide a coherent framework to define the frames of reference for the interpretation of reference recommendations [29]. The population (P) was (adapted from a previous similar study [29]) for whom the recommendations from either the 2017 ACC/AHA guideline [22] or the 2018 ESC/ESH guideline [23] were intended; the intervention (I) was defined as the standard approach adopted in either the 2017 ACC/AHA guideline [22] or the 2018 ESC/ESH guideline [23]; whereas the comparator (C) was defined as the approach in contrast to the standard approach specified in either the 2017 ACC/AHA guideline [22] or the 2018 ESC/ESH guideline [23].

### Coding of reference recommendations

For each reference recommendation, a coder, who was a registered primary care pharmacist actively practicing in the management of hypertension, compared and coded the recommendation from each of the included CPGs from Southeast Asia in two steps. First, the coder determined whether a given reference recommendation was adequately addressed in the included CPGs from Southeast Asia to allow for concordance mapping; if it was not adequately addressed, the recommendation was marked ‘out of scope’ for that CPG and ruled out from further analysis. Second, each in-scope reference recommendation was coded as:

- ‘for’ if the CPG from Southeast Asia recommended the intervention (I) over the comparator (C),
- ‘against’ if the CPG from Southeast Asia recommended the comparator (C) over the intervention (I),
- ‘insufficient’ if the CPG from Southeast Asia did not recommend ‘for’ or ‘against’ the intervention (I) due to insufficient evidence, but the P-I-C specification was in-scope, or
- ‘different’ if the assertion from the CPG from Southeast Asia could not be classified as ‘for’, ‘against’, or ‘insufficient’.

A code reviewer, who was a registered clinical pharmacist with clinical experience in the management of hypertension, checked the codings of the coder for accuracy. Subsequently, an investigator with academic background reviewed the codings, and any discrepancy identified was resolved by consensus. Final codings were confirmed when a full consensus was achieved.

After confirmation of the codings for every reference recommendation, rate of concordance was calculated. CPG labelled as ‘out of scope’ or coded ‘different’ for a given reference recommendation was not considered for the analyses of the rate of concordance as this indicated an absence of recommendation instead of concordance of recommendation. The reference recommendation was included for the calculation of rate of concordance only if ≥ 2 CPGs from Southeast Asia were coded as ‘for’, ‘against’, or ‘insufficient’ for a given reference recommendation.

For assessments of rate of concordance, the included CPGs were considered to be concordant with either the 2017 ACC/AHA guideline [22] or the 2018 ESC/ESH guideline [23] for a given reference recommendation if all comparator CPG codings were ‘for’, or if all comparator CPG codings were ‘for’ or ‘insufficient’ but ≥ 60% were ‘for’. On the other hand, the included CPGs was regarded as non-concordant with either the 2017 ACC/AHA guideline [22] or the 2018 ESC/ESH guideline [23] for a given reference recomendation if any comparator CPG coding was ‘for’ and any other comparator CPG coding was ‘against’, if comparator CPG codings were all either ‘against’ or ‘insufficient’, or if all comparator CPG codings were ‘for’ or ‘insufficient’ but < 60% were ‘for’.

### Sensitivity analysis

Sensitivity analysis was conducted by the exclusion of ‘insufficient’ ratings from the analyses of rate of concordance. In addition, a leave-one-out sensitivity analysis was also performed to assess rates of concordance with the exclusion of each included CPG one at a time.

## Results

### Characteristics of included clinical practice guidelines

We identified six CPGs [30–35] that corresponded to our inclusion and exclusion criteria, each one of them originated from Thailand, Malaysia, Indonesia, Brunei, Singapore, and Vietnam. We have formally approached the relevant professional bodies of the remaining five countries (the Philippines, Laos, Cambodia, Myanmar, and Timor Leste), but we were told that they did not produce CPGs for the management of hypertension at the time of conducting this study.

Table 1 displays the characteristics of the included CPGs originated from Southeast Asia [30–35]. All the included CPGs [30–35] were published within the last five years (2017–2020). Except for the two CPGs originated from Malaysia [30] and Singapore [32] respectively which were developed by governmental institution, the remaining four CPGs [31, 33–35] were either developed by cardiovascular specialty societies or through joint collaboration of governmental institution and cardiovascular specialty societies.

**Table 1:** Characteristics of the included clinical practice guidelines

Characteristic	Malaysia [30]	Brunei [31]	Singapore [32]	Thailand [33]	Indonesia [34]	Vietnam [35]
Year of publication	2018	2019	2017	2019	2019	2018
Status of the CPG	Update from 2013 version	Update from 2002 version	Update from 2005 version	Update from 2015 version	Update from 2014 version	Update from 2015 version
Type of elaboration organization	Governmental institution	Joint collaboration of governmental institution and specialty society	Governmental institution	Specialty society	Specialty society	Specialty society
Funding/sponsorship	Industry educational grant	Not reported	Not reported	Not reported	Not reported	Not reported
No. of GDG members	19	20	15	15	18	23
Total no. of references cited	506	127	137	99	35	Not reported
Total no. of systematic reviews cited	64	4	9	8	3	Not reported
Total no. of Cochrane reviews cited	20	2	2	0	1	Not reported
Evidence classification method	SIGN adapted	No classification	GRADE adapted	Own method	No classification	Own method

CPG: clinical practice guideline; GDG: guideline development group; GRADE: Grading of Recommendations, Assessment, Development, and Evaluations; SIGN: Scottish Intercollegiate Guidelines Network

### Generation of reference recommendations

A total of 59 reference recommendations with unique and unambiguous P-I-C specifications was generated from the 2017 ACC/AHA guideline [22]. In addition, a total of 51 reference recommendations with unique and unambiguous P-I-C specifications was generated from the 2018 ESC/ESH guideline [23]. Table 2 depicts the P-I-C specifications of the reference recommendations from the 2017 ACC/AHA guideline (starts with the code ACC) and the 2018 ESC/ESH guideline (starts with the code ESC), respectively. These reference recommendations can be classified into seven different sections: “blood pressure measurement” (ACC-1; ESC-1), “diagnosis of hypertension” (ACC-2 to ACC-6; ESC-2 to ESC-5), “investigations in patients with hypertension” (ACC-7 to ACC-17; ESC-6 to ESC-15), “lifestyle modifications” (ACC-18 to ACC-23; ESC-16 to ESC-21), “goal blood pressure” (ACC-24 to ACC-31; ESC-22 to ESC-28), “pharmacotherapy for patients with hypertension and no comorbidity” (ACC-32 to ACC-41; ESC-29 to ESC-38), and “pharmacotherapy for patients with hypertension and comorbidity” (ACC-42 to ACC-59; ESC-39 to ESC-51). Full descriptions of the 59 reference recommendations generated from the 2017 ACC/AHA guideline and the 51 reference recommendations generated from the 2018 ESC/ESH guideline can be found in the supplementary files (Additional file 1: Table S1).

**Table 2:** Population-intervention-comparator specifications of reference recommendations

Code^1^	Population	Intervention	Comparator
ACC-1 ESC-1	All patients	Blood pressure measurement with specific instructions on cuff size and body position of patients	Blood pressure measurement without specific instructions
ACC-2 ESC-2	Adults with suspected hypertension	Diagnosis based on at least 2 blood pressure measurements per office visit from at least 2 office visits	Diagnosis based on single blood pressure measurement and/or single office visit
ACC-3	Adults with suspected hypertension	Diagnosis based on the office (non-automated) systolic blood pressure measurement of > 130 mm Hg and/or diastolic blood pressure measurement of > 80 mm Hg	Diagnosis based on thresholds other than systolic blood pressure measurement of > 130 mm Hg and/or diastolic blood pressure measurement of > 80 mm Hg
ESC-3	Adults with suspected hypertension	Diagnosis based on the office (non-automated) systolic blood pressure measurement of > 140 mm Hg and/or diastolic blood pressure measurement of > 90 mm Hg	Diagnosis based on thresholds other than systolic blood pressure measurement of > 140 mm Hg and/or diastolic blood pressure measurement of > 90 mm Hg
ACC-4	Adults with suspected hypertension but without diagnostic uncertainty and blood pressure variability	Ambulatory blood pressure monitoring to confirm the diagnosis of hypertension	Diagnosis based on office blood pressure measurements alone
ACC-5	Adults with suspected hypertension but without diagnostic uncertainty and blood pressure variability	Home blood pressure monitoring to confirm the diagnosis of hypertension	Diagnosis based on office blood pressure measurements alone
ACC-6 ESC-4	Adults with suspected hypertension but with diagnostic uncertainty	Performance of ambulatory blood pressure monitoring to confirm the diagnosis of hypertension	No performance of ambulatory blood pressure monitoring to confirm the diagnosis of hypertension
ESC-5	Adults with suspected blood pressure variability	Performance of ambulatory blood pressure monitoring to confirm the diagnosis of hypertension	No performance of ambulatory blood pressure monitoring to confirm the diagnosis of hypertension
ACC-7 ESC-6	Adults newly diagnosed with hypertension	Performance of baseline blood chemistry (sodium, potassium, creatinine)	No performance of baseline blood chemistry
ACC-8 ESC-7	Adults newly diagnosed with hypertension	Performance of baseline fasting blood glucose level test	No performance of baseline fasting blood glucose level test
ACC-9 ESC-8	Adults newly diagnosed with hypertension	Performance of baseline fasting lipid profile test	No performance of baseline fasting lipid profile test
ACC-10 ESC-9	Adults newly diagnosed with hypertension	Performance of baseline dipstick urinalyis for the detection of blood and protein	No performance of baseline dipstick urinalyis
ACC-11 ESC-10	Adults newly diagnosed with hypertension	Performance of baseline electrocardiography	No performance of baseline electrocardiography
ACC-12 ESC-11	Adults newly diagnosed with hypertension	Performance of baseline serum hemoglobin or hematocrit level test	No performance of baseline serum hemoglobin or hematocrit level test
ACC-13	Adults newly diagnosed with hypertension	Performance of baseline serum calcium level test	No performance of baseline serum calcium level test
ACC-14 ESC-12	Adults newly diagnosed with hypertension	Performance of baseline serum uric acid level test	No performance of baseline serum uric acid level test
ESC-13	Adults newly diagnosed with hypertension	Performance of urine testing for albumin: creatinine ratio to quantify baseline albumin level	No performance of urine testing to quantify baseline albumin level
ACC-15	Adults newly diagnosed with hypertension	Performance of urine testing for albumin: creatinine ratio to quantify baseline albumin level	Performance of 24-h urine testing to quantify baseline albumin level
ACC-16 ESC-14	Adults newly diagnosed with hypertension	Targeted screening for potential underlying causes of secondary hypertension	No screening for potential underlying causes of secondary hypertension
ACC-17 ESC-15	Adults newly diagnosed with hypertension and with suspected structural heart disease	Performance of baseline echocardiography	No performance of baseline echocardiography
ACC-18 ESC-16	Adults with hypertension and concurrent overweight/obesity	Counseling for weight loss	No counseling for weight loss
ACC-19 ESC-17	Adults with hypertension	Counseling for dietary modifications	No counseling for dietary modifications
ACC-20 ESC-18	Adults with hypertension	Counseling for regular physical activity	No counseling for regular physical activity
ACC-21 ESC-19	Adults with hypertension who smoke	Counseling for smoking cessation	No counseling for smoking cessation
ACC-22 ESC-20	Adults with hypertension	Counseling for salt/sodium restriction	No counseling for salt/sodium restriction
ACC-23 ESC-21	Adults with hypertension and heavy alcohol consumption	Counseling to moderate alcohol consumption	No counseling on alcohol consumption
ACC-24	Adults age 18–60 years with hypertension but without diabetes, coronary artery disease, and chronic kidney disease	Goal blood pressure of < 130/80 mm Hg	Other goal blood pressure
ESC-22	Adults age 18–60 years with hypertension but without diabetes, coronary artery disease, and chronic kidney disease	Goal blood pressure of < 140/90 mm Hg	Other goal blood pressure
ACC-25	Adults age 60–80 years with hypertension but without diabetes, coronary artery disease, and chronic kidney disease	Goal blood pressure of < 130/80 mm Hg	Other goal blood pressure
ESC-23	Adults age 60–80 years with hypertension but without diabetes, coronary artery disease, and chronic kidney disease	Goal blood pressure of < 140/90 mm Hg	Other goal blood pressure
ACC-26	Adults age > 50 years with increased risk of cardiovascular disease	Goal systolic blood pressure of < 130 mm Hg	Other goal systolic blood pressure
ACC-27	Adults age > 75–80 years with hypertension	Goal blood pressure of < 130/80 mm Hg	Higher goal blood pressure
ESC-24	Adults age > 75–80 years with hypertension	Goal blood pressure of < 140/90 mm Hg	Other goal blood pressure
ACC-28	Adults with hypertension and concurrent diabetes	Goal blood pressure of < 130/80 mm Hg	Other goal blood pressure
ESC-25	Adults with hypertension and concurrent diabetes	Goal blood pressure of < 140/80 mm Hg	Other goal blood pressure
ACC-29	Adults with hypertension and concurrent chronic kidney disease but without proteinuria and diabetes	Goal blood pressure of < 130/80 mm Hg	Other goal blood pressure
ESC-26	Adults with hypertension and concurrent chronic kidney disease but without proteinuria and diabetes	Goal blood pressure of < 140/90 mm Hg	Other goal blood pressure
ACC-30 ESC-27	Adults with hypertension and concurrent chronic kidney disease with proteinuria	Goal blood pressure of < 130/80 mm Hg	Other goal blood pressure
ACC-31	Adults with hypertension and concurrent chronic kidney disease and diabetes	Goal blood pressure of < 130/80 mm Hg	Other goal blood pressure
ESC-28	Adults with hypertension and concurrent chronic kidney disease and diabetes	Goal blood pressure of < 140/90 mm Hg	Other goal blood pressure
ACC-32 ESC-29	Adults age < 60 years with hypertension but with no comorbidity requiring initial pharmacotherapy	Thiazide diuretics as an option for first-line therapy	Not recommending thiazide diuretics as an option for first-line therapy
ACC-33 ESC-30	Adults age ≥ 60 years with hypertension but with no comorbidity requiring initial pharmacotherapy	Thiazide diuretics as an option for first-line therapy	Not recommending thiazide diuretics as an option for first-line therapy
ACC-34 ESC-31	Adults age < 60 years with hypertension but with no comorbidity requiring initial pharmacotherapy	ACE inhibitors as an option for first-line therapy	Not recommending ACE inhibitors as an option for first-line therapy
ACC-35 ESC-32	Adults age ≥ 60 years with hypertension but with no comorbidity requiring initial pharmacotherapy	ACE inhibitors as an option for first-line therapy	Not recommending ACE inhibitors as an option for first-line therapy
ACC-36 ESC-33	Adults age < 60 years with hypertension but with no comorbidity requiring initial pharmacotherapy	ARBs as an option for first-line therapy	Not recommending ARBs as an option for first-line therapy
ACC-37 ESC-34	Adults age ≥ 60 years with hypertension but with no comorbidity requiring initial pharmacotherapy	ARBs as an option for first-line therapy	Not recommending ARBs as an option for first-line therapy
ACC-38 ESC-35	Adults age < 60 years with hypertension but with no comorbidity requiring initial pharmacotherapy	Calcium channel blockers as an option for first-line therapy	Not recommending calcium channel blockers as an option for first-line therapy
ACC-39 ESC-36	Adults age ≥ 60 years of age with hypertension but with no comorbidity requiring initial pharmacotherapy	Calcium channel blockers as an option for first-line therapy	Not recommending calcium channel blockers as an option for first-line therapy
ACC-40	Adults with hypertension but with no comorbidity requiring initial pharmacotherapy	Not recommending beta-blockers as an option for first-line therapy	Beta-blockers as an option for first-line therapy
ESC-37	Adults with hypertension but with no comorbidity requiring initial pharmacotherapy	Beta-blockers as an option for first-line therapy	Not recommending beta-blockers as an option for first-line therapy
ACC-41 ESC-38	Adults with hypertension but with no comorbidity requiring initial pharmacotherapy	Thiazide diuretics, ACE inhibitors, ARBs, or calcium channel blockers are preferred over beta-blockers as an option for first-line therapy	Beta-blockers as equally preferred option as thiazide diuretics, ACE inhibitors, ARBs, and calcium channel blockers for first-line therapy
ACC-42 ESC-39	Adults with hypertension and concurrent diabetes	ACE inhibitors or ARBs as an option for first-line therapy	Not recommending ACE inhibitors or ARBs as an option for first-line therapy
ACC-43	Adults with hypertension and concurrent diabetes	ACE inhibitors, ARBs, calcium channel blockers, or thiazide diuretics as the preferred option for first-line therapy	Other drug therapies are preferred over ACE inhibitors, ARBs, calcium channel blockers, or thiazide diuretics as an option for first-line therapy
ACC-44 ESC-40	Adults with hypertension and concurrent chronic kidney disease	ACE inhibitors as an option for first-line therapy	Not recommending ACE inhibitors as an option for first-line therapy
ACC-45 ESC-41	Adults with hypertension and concurrent chronic kidney disease	ARBs as equally preferred option as ACE inhibitors for first-line therapy	ACE inhibitors are preferred over ARBs as an option for first-line therapy
ACC-46	Adults with hypertension and concurrent chronic kidney disease but without moderately increased albuminuria	Thiazide diuretics or calcium channel blockers as equally preferred option as ACE inhibitors or ARBs for first-line therapy	ACE inhibitors or ARBs are preferred over thiazide diuretics or calcium channel blockers as an option for first-line therapy
ACC-47 ESC-42	Adults with hypertension and concurrent chronic kidney disease with at least moderately increased albuminuria	ACE inhibitors or ARBs as the preferred option for first-line therapy	Drug therapies other than ACE inhibitors or ARBs as equally or more preferred option for first-line therapy
ACC-48	Adults with hypertension and concurrent chronic kidney disease intolerant to ACE inhibitors	ARBs as an option for first-line therapy	Not recommending ARBs as an option for first-line therapy
ACC-49	Adults with hypertension and concurrent chronic kidney disease intolerant to ACE inhibitors	ARBs as the preferred option for first-line therapy	Drug therapies other than ARBs as equally or more preferred option for first-line therapy
ACC-50 ESC-43	Adults with hypertension and concurrent coronary artery disease (ischemic heart disease) but without prior acute coronary syndrome	ACE inhibitors as an option for first-line therapy	Not recommending ACE inhibitors as an option for first-line therapy
ACC-51	Adults with hypertension and prior acute coronary syndrome	ACE inhibitors as an option for first-line therapy	Not recommending ACE inhibitors as an option for first-line therapy
ACC-52 ESC-44	Adults with hypertension and concurrent coronary artery disease (ischemic heart disease) but without prior acute coronary syndrome	ARBs as equally preferred option as ACE inhibitors for first-line therapy	ACE inhibitors are preferred over ARBs as an option for first-line therapy
ACC-53	Adults with hypertension and prior acute coronary syndrome	ARBs as equally preferred option as ACE inhibitors for first-line therapy	ACE inhibitors are preferred over ARBs as an option for first-line therapy
ESC-45	Adults with hypertension and concurrent coronary artery disease (ischemic heart disease) but without prior acute coronary syndrome	Beta-blockers or calcium channel blockers as equally or more preferred option than ACE inhibitors or ARBs for first-line therapy	ACE inhibitors or ARBs are preferred over beta-blockers or calcium channel blockers as an option for first-line therapy
ESC-46	Adults with hypertension and prior acute coronary syndrome	Beta-blockers, ACE inhibitors, or ARBs as the preferred option for first-line therapy	Other drug therapies as equally or more preferred option than beta-blockers, ACE inhibitors, or ARBs for first-line therapy
ACC-54 ESC-47	Adults with hypertension and concurrent coronary artery disease (ischemic heart disease) but without prior acute coronary syndrome intolerant to ACE inhibitors	ARBs as an option for first-line therapy	Not recommending ARBs as an option for first-line therapy
ACC-55	Adults with hypertension and prior acute coronary syndrome intolerant to ACE inhibitors	ARBs as an option for first-line therapy	Not recommending ARBs as an option for first-line therapy
ESC-48	Adults with hypertension and concurrent coronary artery disease (ischemic heart disease) but without prior acute coronary syndrome intolerant to ACE inhibitors	Beta-blockers or calcium channel blockers as the preferred option for first-line therapy	ARBs as equally or more preferred option than beta-blockers or calcium channel blockers for first-line therapy
ACC-56 ESC-49	Adults with hypertension and recent acute coronary syndrome	Beta-blockers as an option for first-line therapy	Not recommending beta-blockers as an option for first-line therapy
ACC-57 ESC-50	Adults with hypertension and concurrent heart failure	ACE inhibitors as an option for first-line therapy	Not recommending ACE inhibitors as an option for first-line therapy
ACC-58 ESC-51	Adults with hypertension and concurrent heart failure	Beta-blockers as an option for first-line therapy	Not recommending beta-blockers as an option for first-line therapy
ACC-59	Adults with hypertension and concurrent heart failure intolerant to ACE inhibitors	ARBs as an option for first-line therapy	Not recommending ARBs as an option for first-line therapy

ACE: Angiotensin-converting enzyme; ARB: Angiotensin II receptor blocker

^1^Reference recommendations with code starting with ACC were generated from the 2017 ACC/AHA hypertension guideline [22] whereas reference recommendations with code starting with ESC were generated from the 2018 ESC/ESH hypertension guideline [23]

### Concordance of recommendations for the management of hypertension with 2017 ACC/AHA guideline

Considering the six included CPGs from Southeast Asia [30–35], concordance was observed for 30 reference recommendations (50.8%) out of 59 reference recommendations generated from the 2017 ACC/AHA guideline [22] (Table 3). The codings of reference recommendations generated from the 2017 ACC/AHA guideline can be found in the supplementary files (Additional file 1: Table S2). Full concordance (100%) was observed for reference recommendations in the section of “blood pressure measurement” (n = 1/1) and in the section of “lifestyle modifications” (n = 6/6) (Table 4). Concordance of reference recommendations in the section of “pharmacotherapy for patients with hypertension and no comorbidity” (n = 6/10) and in the section of “pharmacotherapy for patients with hypertension and comorbidity” (n = 12/18) achieved rates of 60.0% and 66.7%, respectively. Reference recommendations in the remaining sections achieved less than half concordance (50%): 20.0% (n = 1/5) in the section of “diagnosis of hypertension” and 36.3% (n = 4/11) in the section of “investigations in patients with hypertension”. Complete non-concordance (0%) was reported for the reference recommendations in the section of “goal blood pressure” (n = 0/8) (Table 4).

**Table 3:** Rate of concordance of recommendations in the primary analysis and sensitivity analysis

	2017 ACC/AHA guideline		2018 ESC/ESH guideline	
	Reference recommendations, No	Concordance, No. (%)	Reference recommendations, No	Concordance, No. (%)
Primary analysis	59	30 (50.8)	51	31 (60.8)
Sensitivity analysis excluding insufficient ratings	55	33 (60.0)	49	32 (65.3)
Sensitivity analysis excluding CPG originated from				
Malaysia	59	30 (50.8)	51	31 (60.8)
Brunei	59	32 (54.2)	51	33 (64.7)
Singapore	59	30 (50.8)	51	31 (60.8)
Thailand	59	31 (52.5)	51	32 (62.7)
Indonesia	59	33 (55.9)	51	34 (66.7)
Vietnam	59	31 (52.5)	51	32 (62.7)

ACC: American College of Cardiology; AHA: American Heart Association; BRN: Brunei; ESC: European Society of Cardiology; ESH: European Society of Hypertension

**Table 4:** Rate of concordance of recommendations according to different sections in the primary analysis and sensitivity analysis

	2017 ACC/AHA guideline		2018 ESC/ESH guideline	
	Reference recommendations, No	Concordance, No. (%)	Reference recommendations, No	Concordance, No. (%)
Primary analysis				
Blood pressure measurement^a^	1	1 (100)	1	1 (100)
Diagnosis of hypertension^b^	5	1 (20.0)	4	3 (75.0)
Investigations of patients with hypertension^c^	11	4 (36.3)	10	4 (40.0)
Lifestyle modifications^d^	6	6 (100)	6	6 (100)
Goal blood pressure^e^	8	0 (0)	7	0 (0)
Pharmacotherapy for patients with hypertension and no comorbidity^f^	10	6 (60.0)	10	6 (60.0)
Pharmacotherapy for patients with hypertension and comorbidity^g^	18	12 (66.7)	13	11 (84.6)
Sensitivity analysis excluding insufficient ratings				
Blood pressure measurement^a^	1	1 (100)	1	1 (100)
Diagnosis of hypertension^b^	5	2 (40.0)	4	4 (100)
Investigations of patients with hypertension^c^	11	5 (45.4)	10	4 (40.0)
Lifestyle modifications^d^	6	6 (100)	6	6 (100)
Goal blood pressure^e^	8	0 (0)	7	0 (0)
Pharmacotherapy for patients with hypertension and no comorbidity^f^	10	6 (60.0)	10	6 (60.0)
Pharmacotherapy for patients with hypertension and comorbidity^g^	14	13 (92.9)	11	11 (100)

ACC: American College of Cardiology; AHA: American Heart Association; ESC: European Society of Cardiology; ESH: European Society of Hypertension

^a^ACC-1; ESC-1

^b^ACC-2 to ACC-6; ESC-2 to ESC-5

^c^ACC-7 to ACC-17; ESC-6 to ESC-15

^d^ACC-18 to ACC-23; ESC-16 to ESC-21

^e^ACC-24 to ACC-31; ESC-22 to ESC-28

^f^ACC-32 to ACC-41; ESC-29 to ESC-38

^g^ACC-42 to ACC-59; ESC-38 to ESC-51

### Concordance of recommendations for the management of hypertension with 2018 ESC/ESH guideline

Considering the six included CPGs from Southeast Asia [30–35], concordance was observed for 31 reference recommendations (69.8%) out of 51 reference recommendations derived from the 2018 ESC/ESH guideline [23] (Table 3). The codings of reference recommendations generated from the 2018 ESC/ESH guideline can be found in the supplementary files (Additional file 1: Table S3). Reference recommendations in the section of “blood pressure measurement” (n = 1/1) and in the section of “lifestyle modifications” (n = 6/6) achieved full concordance (100%) (Table 4). This was followed by concordance in the reference recommendations consist in the section of “pharmacotherapy for patients with hypertension and comorbidity” (n = 11/13), in the section of “diagnosis of hypertension” (n = 3/4), and in the section on “pharmacotherapy for patients with hypertension and no comorbidity” (n = 6/10), which achieved rates of 84.6%, 75.0%, and 60.0%, respectively (Table 4). Reference recommendations in the remaining sections achieved less than half concordance (50%): 40.0% (n = 4/10) in the section of “investigations in patients with hypertension” and 0% (n = 0/7) in the section of “goal blood pressure” (Table 4).

### Sensitivity analysis excluding insufficient ratings

There was no substantial change in the rate of concordance when “insufficient” ratings were removed from consideration in the sensitivity analysis (Table 3, Additional file 1: Table S2, and Table S3). Since four reference recommendations (ACC-48, ACC-49, ACC-56, and ACC-57) in the 2017 ACC/AHA guideline [22] and two reference recommendations (ESC-49, and ESC-50) in the 2018 ESC/ESH guideline [23], respectively, were with insufficient ratings for all of the six included CPGs, only 55 reference recommendations from the 2017 ACC/AHA guideline and 49 reference recommendations from the 2018 ESC/ESH guideline were considered in this sensitivity analysis. Across the six included CPGs [95–100], concordance was found for 33 reference recommendations (60.0%) out of 55 reference recommendations identified from the 2017 ACC/AHA guideline [26]. Whereas concordance was found for 32 reference recommendations (65.3%) out of 49 reference recommendations identified from the 2018 ESC/ESH guideline [27].

### Leave-one-out sensitivity analysis

The findings from the leave-one-out sensitivity analyses indicated that no single CPG could explain the non-concordance of recommendations (Table 3, Additional file 1: Table S4, and Table S5). The rate of concordance of recommendations in the 2017 ACC/AHA guideline [22] changed by an absolute of 0% (with the removal of either the CPG originated from Malaysia [30] or the CPG originated from Singapore [32]), through 1.7% (with the removal of either the CPG originated from Thailand [33] or the CPG originated from Vietnam [35]) and 3.4% (with the removal of the CPG originated from Brunei [31]), to 5.1% (with the removal of the CPG originated from Indonesia [34]) in the leave-one-out sensitivity analyses. Likewise, the rate of concordance of recommendations in the 2018 ESC/ESH guideline [23] changed by an absolute of 0% (with the removal of either the CPG originated from Malaysia [30] or the CPG originated from Singapore [32]), through 1.9% (with the removal of either the CPG originated from Thailand [33] or the CPG originated from Vietnam [35]) and 3.9% (with the removal of the CPG originated from Brunei [31]), to 5.9% (with the removal of the CPG originated from Indonesia [34]) in the leave-one-out sensitivity analyses.

### Sources of non-concordance in recommendations in the management of hypertension from the 2017 ACC/AHA guideline

Across the six included CPGs from Southeast Asia [30–35], non-concordance was observed for 29 reference recommendations (49.2%) out of 59 reference recommendations generated from the 2017 ACC/AHA guideline [22]. The majority of the non-concordant recommendations were in the section of “goal blood pressure”, where none of the eight reference recommendations in this section were concordant across the included CPGs [30–35]. Specifically, the included CPGs [30–35] were non-concordant for the goal blood pressure  (130/80 mm Hg or lower) specified for adults (for the age groups of 18–60 years [ACC-24], 60–80 years [ACC-25], and > 75–80 years [ACC-27]) with hypertension but with no comorbidity in the 2017 ACC/AHA guideline [26]. In addition, the included CPGs [30–35] were non-concordant for the goal blood pressure (130/80 mm Hg or lower) specified for adults with hypertension and comorbidity (diabetes [ACC-28] or chronic kidney disease [ACC-29 and ACC-30] or both [ACC-31]) in the 2017 ACC/AHA guideline [22].

Seven out of eleven reference recommendations in the section of “investigations of patients with hypertension” were non-concordant across the included CPGs [95–100]. Specifically, included CPGs [30–35] were non-concordant for the recommendations to perform baseline blood chemistry (sodium, potassium, creatinine) (ACC-7), fasting blood glucose level test (ACC-8), fasting lipid profile test (ACC-9), serum hemoglobin or haematocrit level test (ACC-12), serum calcium level test (ACC-13), serum uric acid level test (ACC-14), and urine testing for albumin: creatinine ratio (ACC-15), in all adults newly diagnosed with hypertension, as specified in the 2017 ACC/AHA guideline [22].

Other sources of non-concordance in recommendations were in the section of “pharmacotherapy for patients with hypertension and comorbidity” (six out of 18 recommendations were non-concordant; Table 4). Included CPGs were non-concordant for the recommendation in the 2017 ACC/AHA guideline [22] to prescribe thiazide diuretics or calcium channel blockers as equally preferred option as angiotensin converting enzyme (ACE) inhibitors or angiotensin II receptor blockers (ARBs) for first-line therapy in adults with hypertension and chronic kidney disease but without moderately increased albuminuria (ACC-46). Other non-concordant recommendations in this section either turned concordant (n = 1; ACC-59) or were no longer in consideration (n = 4; ACC-48, ACC-49, ACC-54, ACC-55) once “insufficient” ratings were removed in the sensitivity analyses.

### Sources of non-concordance in recommendations in the management of hypertension from the 2018 ESC/ESH guideline

Across the six included CPGs from Southeast Asia [30–35], non-concordance was observed for 20 reference recommendations (39.2%) out of 51 reference recommendations generated from the 2018 ESC/ESH guideline [23]. Likewise, the largest sources of non-concordant recommendations were in the section of “goal blood pressure”, where none of the seven reference recommendations in this section were concordant across the included CPGs [30–35]. Specifically, the included CPGs [30–35] were non-concordant for the goal blood pressure specified for adults (for the age groups of 18–60 years [ESC-22], 60–80 years [ESC-23], and > 75–80 years [ESC-24]) with hypertension but with no comorbidity in the 2018 ESC/ESH guideline [23]. In addition, the included CPGs [30–35] were non-concordant for the goal blood pressure specified for adults with hypertension and comorbidity (diabetes [ESC-25] or chronic kidney disease [ESC-26 and ESC-27] or both [ESC-28]) in the 2018 ESC/ESH guideline [23].

The second largest sources of non-concordant reference recommendations were in the section of “investigations of patients with hypertension”, with six out of ten non-concordant recommendations across the included CPGs [30–35]. Specifically, included CPGs [30–35] were non-concordant for the recommendations to perform baseline blood chemistry (sodium, potassium, creatinine) (ESC-6), fasting blood glucose level test (ESC-7), fasting lipid profile test (ESC-8), serum hemoglobin or haematocrit level test (ESC-11), serum uric acid level test (ESC-12), and urine testing for albumin: creatinine ratio (ESC-13) in all adults newly diagnosed with hypertension as specified in the 2018 ESC/ESH guideline [23].

Other sources of non-concordance in recommendations were in the section of “pharmacotherapy for patients with hypertension and no comorbidity” (n = 4) which included the recomendations of thiazide diuretics (ESC-29 and ESC-30) and beta-blocker (ESC-37) as an option for first-line therapy, as well as the recommendation of thiazide diuretics, ACE inhibitors, ARBs, or calcium channel blockers being preferred over beta-blockers as an option for first-line therapy (ESC-38), in adults with hypertension but with no comorbidity requiring initial pharmacotherapy.

## Discussion

It was encouraging to observe the concordance of the recommendations for proper blood pressure measurement and lifestyle modifications across the CPGs for the management of hypertension in Southeast Asia [30–35] with the 2017 ACC/AHA guideline [22] and the 2018 ESC/ESH guideline [23]. Accurate measurement and recording of blood pressure are of utmost importance in order to accurately classify the level of blood pressure, to guide management of hypertension, and to ascertain blood-pressure-related cardiovascular risk [22]. Although measurement of blood pressure in the office settings is relatively easy, it is not redundant for the emphasis in the CPGs for proper techniques of measurement, since in real-world clinical practice, it is often performed without adequate attention to the specified preconditions required for a valid measurement, which could lead to misestimation of patients’ true level of blood pressure and prescription of unnecessary treatment [22, 23]. On the other hand, nonpharmacological lifestyle interventions are effective in lowering blood pressure for patients with hypertension, with the most important approaches being weight loss, sodium reduction, increased physical activity, increased consumption of vegetables and fruits, reduction in alcohol consumption, and smoking cessation [22, 23]. The recommendations of these lifestyle interventions were consistent across CPGs for the management of hypertension in Southeast Asia [30–35], and thus complete concordance with the 2017 ACC/AHA guideline [22] and the 2018 ESC/ESH guideline [23].

The blood pressure targets for patients with hypertension had always been controversial since the publication of the 2017 ACC/AHA guideline [22] which issued a groundbreaking recommendation that the goal blood pressure for most of the patients with hypertension should be < 130/80 mm Hg, including those without comorbidity. However, the 2018 ESC/ESH guideline [23] did not concur with the 2017 ACC/AHA guideline where a primary goal blood pressure of < 140/90 mm Hg was still recommended for all patients with hypertension but without comorbidity. Likewise, the recommended blood pressure target for patients with hypertension but without comorbidity was divided across the CPGs for the management of hypertension in Southeast Asia. Specifically, the CPGs originated from Malaysia [30], Brunei [31], and Singapore [32] respectively, were concordant with the 2018 ESC/ESH guideline [27], and the CPGs originated from Thailand [33], Indonesia [34], and Vietnam [35] respectively, were concordant with the 2017 ACC/AHA guideline [22], on the goal blood pressure for patients with hypertension age 18–60 years without comorbidity. None of the CPGs for the management of hypertension in Southeast Asia [30–35] was concordant with the 2017 ACC/AHA guideline [22] on the goal  blood pressure (< 130/80 mm Hg) for patients with hypertension age > 60 years but without comorbidity, but the CPGs originated from Malaysia [30], Brunei [31], Singapore [32], and Thailand [33], respectively, were concordant with the 2018 ESC/ESH guideline [23] on the goal blood pressure (< 140/90 mm Hg) for patients with hypertension age 60–80 years but without comorbidity. CPGs originated from Indonesia [34] and Vietnam [35] respectively, advocated different blood pressure targets (< 140/80 mm Hg) than that specified in either the 2017 ACC/AHA guideline [22] or the 2018 ESC/ESH guideline [23]. Nonetheless, these CPGs from Southeast Asia [30–35] did not provide the rationale as to their recommended blood pressure targets in these patient populations.

Undeniably, the totality of the existing evidence in patients with hypertension indicates a reduction in the risk of major cardiovascular events and cardiovascular mortality with more intensive blood pressure lowering relative to standard blood pressure lowering. Specifically, the systematic review and meta-analysis (n = 23,169) [36] performed to inform the 2017 ACC/AHA guideline [22], which included randomized controlled trials with a systolic blood pressure target of < 130 mm Hg compared with any higher systolic blood pressure target reported significant risk reduction for stroke (relative risk = 0.82; 95% CI 0.70–0.96) and major cardiovascular events (relative risk = 0.84; 95% confidence interval 0.73–0.99). Similarly, another meta-analysis [37] of all available randomized controlled trials (n = 613,815) which had been cited in the 2018 ESC/ESH guideline [23] observed that further reduction per 10 mm Hg in systolic blood pressure reduced the rate of major cardiovascular events and death, even in patients with baseline systolic blood pressure between 130 and 139 mm Hg, indicating benefit at achieved systolic blood pressure of < 130 mm Hg. However, a meta-analysis of randomized trials (n = 255,70) [38] also reported that permanent discontinuation of drug therapy owing to adverse effects was significantly higher in patients with hypertension who had been targeted to achieve lower blood pressure. Therefore, advocating more intensive blood pressure lowering has to be considered alongside the accompanying risk of treatment discontinuation due to adverse events, which may counterweigh the limited incremental risk reduction of major cardiovascular events, and such consideration was the rationale that the 2018 ESC/ESH guideline [23] still recommended a primary blood pressure target of < 140/90 mm Hg. The recommendations of goal blood pressure across the included CPGs of Southeast Asia [30–35] most probably did not consider the cost-effectiveness of different goals blood pressure; in order to better inform the clinical practice, the CPG developer groups should conduct local cost-effectiveness analyses to determine if more intensive blood pressure lowering relative to standard blood pressure lowering is cost-effective, to balance between potential cost saving associated with an incremental reduction in major cardiovascular events and additional cost that would be spent for clinical care used to maintain lower blood pressure, including treatment for adverse events.

CPGs for the management of hypertension in Southeast Asia [30–35] were concordant with the recommendations in both the 2017 ACC/AHA guideline [26] and the 2018 ESC/ESH guideline [27] that ACE inhibitors, ARBs, and calcium channel blockers as the options for initial first-line therapy for patients with hypertension and no comorbidity. These three classes of antihypertensive agents have proven ability to reduce blood pressure and cardiovascular events, with broad equivalence on the risk reduction of overall cardiovascular morbidity and mortality in meta-analyses [37, 39]. However, CPGs for the management of hypertension in Southeast Asia [30–35] were non-concordant with the recommendation of thiazide diuretics as an option for first-line therapy in the said population as specified in both the 2017 ACC/AHA guideline [22] and the 2018 ESC/ESH guideline [23]. The non-concordance was stemmed from the CPG originated from Brunei [31] which did not consider thiazide diuretics as an option for first-line therapy without rationale provided for their exclusion (thiazide diuretics as an option for second-line therapy); the remaining CPGs in Southeast Asia [30, 32–35] listed thiazide-type diuretics as one of the first-line options.

Nonetheless, a systematic review and meta-analysis (n = 247,006) [39] of head-to-head trials of various classes of antihypertensive agents found that the effects of all classes of antihypertensive agents (thiazide diuretics, calcium channel blockers, ACE inhibitors, and ARBs) were not significantly different on all evaluated outcomes, including the risks of stroke, cardiovascular disease, heart failure, cardiovascular death, and all-cause death, when their achieved blood pressure was equivalent. Indeed, thiazide diuretics were superior compared to all other classes of antihypertensive agents to reduce the risk of heart failure in patients with hypertension (relative risk = 0.83; 95% confidence interval 0.73–0.94) [39]. Likewise, the systematic review and meta-analysis (n = 152,379) [36] performed to inform the 2017 ACC/AHA guideline [22] which included head-to-head trials of different classes of antihypertensive agents reported that no other classes of antihypertensive agents (ie, calcium channel blockers, ACE inhibitors, and ARBs) were significantly better than thiazide diuretics as the first-line therapy for the following evaluated outcomes: thiazide diuretics were associated with a significantly lower risk for heart failure relative to calcium channel blockers; significantly lower risk for cardiovascular events and stroke relative to ACE inhibitors; and significantly lower risk for cardiovascular events relative to calcium channel blockers.

Whether beta-blockers should be included as one of the options for initial first-line therapy for patients with hypertension but without comorbidity is still debatable, with divided recommendations between the 2017 ACC/AHA guideline [22] and the 2018 ESC/ESH guideline [23]. The 2017 ACC/AHA guideline [22] did not include beta-blockers as one of the options for first-line therapy, which was followed suit in the CPGs originated from Brunei [31], Indonesia [34], and Vietnam [35], respectively; whereas the 2018 ESC/ESH guideline [23] included beta-blockers as one of the options for first-line therapy, which was followed suit in the CPGs originated from Malaysia [30], Singapore [32], and Thailand [33]. The CPG originated from Malaysia [30] particularly cited a 2017 systematic review [40] which reported that beta-blockers are effective in patients with hypertension < 60 years of age in terms of preventing death, stroke, or myocardial infarction (versus placebo and other antihypertensive agents) and thus they are highly reasonable first-line options in the treatment of hypertension for this population, although the CPG originated from Malaysia [30] itself did not specify the age of patients which beta-blockers should be listed as one of the first-line options. The CPG originated from Thailand [33] though acknowledged that beta-blockers may be inferior to other antihypertensive agents to reduce the risk of cardiovascular diseases, beta-blockers were still being listed as one of the options for first-line therapy due to their similar effects on blood pressure-lowering with other established first-line antihypertensive agents, including ACE inhibitors, ARBs, calcium channel blockers, and thiazide diuretics. The remaining CPGs in Southeast Asia [31, 32, 34, 35] did not provide a rationale for the inclusion or exclusion of beta-blockers as one of the options for first-line therapy.

The notion in the 2018 ESC/ESH guideline [23] that mortality and major cardiovascular outcomes were broadly similar with initial therapy using beta-blockers compared to other first-line antihypertensive agents including ACE inhibitors, ARBs, calcium channel blockers, and thiazide diuretics may not hold true with the currently available evidence. The meta-analysis [39] cited in the 2018 ESC/ESH guideline to justify the recommendation of beta-blockers as one of the initial first-line options for patients with hypertension but without comorbidity has been updated recently. The updated meta-analysis (n = 165,850) [41] which included hypertension trials reported significantly increased risks of stroke (relative risk = 1.21; 95% confidence interval 1.07–1.38), composite of stroke and cardiovascular diseases (relative risk = 1.09; 95% confidence interval 1.01–1.17), and all-cause mortality (relative risk = 1.06; 95% confidence interval 1.01–1.12) with beta-blockers as compared to other first-line antihypertensive agents. Nonetheless, although beta-blockers were listed as one of the options for first-line therapy in the 2018 ESC/ESH guideline, thiazide diuretics, ACE inhibitors, ARBs, and calcium channel blockers were being preferred over beta-blockers as first-line therapy for patients with uncomplicated hypertension [23].

The blood pressure cutoffs for the diagnosis of hypertension was perhaps the most robust debate in the domain of hypertension over the recent years; the 2017 ACC/AHA guideline [22] recommended diagnosis of hypertension based on the office (non-automated) systolic blood pressure reading of ≥ 130 mm Hg and/or diastolic blood pressure reading of ≥ 80 mm Hg, but the 2018 ESC/ESH guideline [23] did not follow suit and recommended the conventional cutoffs based on systolic blood pressure reading of ≥ 140 mm Hg and/or diastolic blood pressure reading of ≥ 90 mm Hg. Interestingly, none of the CPGs for the management of hypertension in Southeast Asia [30–35] followed the cutoffs recommended in the 2017 ACC/AHA guideline [22], including those [30, 31, 33–35] which are published later than the 2017 ACC/AHA guideline [22]; the conventional cutoffs (≥ 140/90 mm Hg) was still being recommended for practice. The developers of the CPG originated from Malaysia [30] believed that the new definition in the 2017 ACC/AHA guideline [22] would not change the way that patients with hypertension was treated, particularly those with cardiovascular complications and blood pressure of ≥ 130/80 mmHg who would need antihypertensive treatment regardless. In addition, the CPG originated from Vietnam [35] believed that the evidence was still insufficient to adopt the new definition recommended in the 2017 ACC/AHA guideline [22].

The notion that the evidence was insufficient with regard to the new cutoffs for diagnosis of hypertension recommended by the 2017 ACC/AHA guideline [22] was probably true since it was merely based on meta-analyses [24, 42–52] of observational data. The 2017 ACC/AHA guideline reviewed the available meta-analyses of observational studies [24, 42–52] and compared the reported hazards for cardiovascular events and stroke of different ranges of blood pressure with a blood pressure of < 120/80 mmHg: patients with a blood pressure of 120–129/80–84 mmHg was similarly at risk for cardiovascular events and stroke, with hazard ratios ranged between 1.1 to 1.5, compared to their counterparts with a blood pressure of 130–139/85–89 mmHg, with hazard ratios ranged between 1.5 to 2.0 [22]. However, the systematic review and meta-analysis [53] of randomized clinical trials with at least 1000 patient-years of follow-up cited in the 2018 ESC/ESH guideline [23] found significant risk reduction of death and cardiovascular events in patients with a baseline blood pressure of ≥ 140/90 mm Hg, while no observed benefits with lower baseline blood pressure. Therefore, it may be prudent to observe the impact in the management of hypertension in the United States of America with the new cutoff recommended in the 2017 ACC/AHA guideline [22] before the introduction of such cutoff in Southeast Asia. Despite not using the new cutoff proposed in the 2017 ACC/AHA guideline [22], all CPGs for the management of hypertension in Southeast Asia [30, 31, 33–35], except the CPG originated from Singapore [32], recommended consideration for antihypertensive drug treatment in patients who have the blood pressure of 130–139/80–89 mm Hg and elevated cardiovascular risk.

Detailed analysis of concordance of recommendations between the CPGs for the management of hypertension in Southeast Asia [30–35] and either the 2017 ACC/AHA guideline [22] or the 2018 ESC/ESH guideline [23] revealed that the justification for the non-concordant recommendations had been poorly described or had not been described at all in the CPGs in Southeast Asia [30–35]. This may be related to a lack of rigor in the construction of CPGs for the management of hypertension in Southeast Asia as previously reported [53]. We believe it might be a worthwhile option for the guideline development groups in Southeast Asia to adapt their recommendations from the existing high-quality CPGs based on formal adaptation frameworks (e.g., GRADE-ADOLOPMENT), as it helps to ensure that their recommendations stay true to the best available evidence while considering the local needs.

This study has some limitations. Firstly, only CPGs for the management of hypertension in Southeast Asia [30–35] published in an official (translated) language of English or Malay were included. Therefore, it was not known if the other versions of the included CPGs published in other official languages (e.g., Thai) had differences content-wise compared to the version of the CPGs published in an official (translated) language of English or Malay. Secondly, we only evaluated the concordance of recommendations in terms of their direction but without considering concordance in terms of their strength of evidence since not all of the included CPGs for the management of hypertension in Southeast Asia [30–35] adopted a formal consensus method to grade the level of evidence and/or strength of the formulated recommendations.

In conclusion, hypertension represents a significant issue that places health and economic strains in Southeast Asia and this demands guideline-based care, yet CPGs for the management of hypertension in Southeast Asia have a high rate of non-concordance with internationally reputable CPGs. Nonetheless, concordant recommendations could perhaps be considered a standard of care for hypertension management in the Southeast Asia region. Conversely, non-concordant recommendations should not be considered a true or stable standard of care, as these represent opposing standards from reputable sources which leave room for flexibility, and clinical autonomy should be used to individualize clinical decisions.

## Supplementary Information

**Additional file 1: Table S1**. Detailed description of reference recommendations. **Table S2**. Concordance of reference recommendations generated from the 2017 ACC/AHA guideline. **Table S3**. Concordance of reference recommendations generated from the 2018 ESC/ESH guideline. **Table S4**. Concordance of reference recommendations generated from the 2017 ACC/AHA guideline after leave-one-out sensitivity analysis. **Table S5**. Concordance of reference recommendations generated from the 2018 ESC/ESH guideline after leave-one-out sensitivity analysis.

## Data Availability

All data generated or analysed during this study are included in this published article and its supplementary information files. Additionally, the clinical practice guidelines analysed during the current study are available in the websites of Academy of Medicine of Malaysia (http://www.acadmed.org.my), Cardiac Society Brunei Darussalam (https://cardiacsociety.org.bn), Ministry of Health, Singapore (https://www.moh.gov.sg), Thai Hypertension Society (http://www.thaihypertension.org), Indonesian Society of Hypertension (https://www.inash.or.id/), and Vietnam National Heart Association (http://www.vnha.org.vn).

## References

[1] World Health Organization (WHO) (2014). Global status report on noncommunicable diseases 2014.

[2] Institute for Public Health (2020). National Health and Morbidity Survey (NHMS) 2019: Non-communicable diseases, healthcare demand, and health literacy—Key Findings.

[3] Kario K, Tomitani N, Buranakitjaroen P (2018). Home blood pressure control status in 2017–2018 for hypertension specialist centers in Asia: results of the Asia BP@Home study. J Clin Hypertens (Greenwich).

[4] Ong SK, Kahan SZ, Lai DTC (2020). Prevalence of undetected hypertension and its association with socio-demographic and non-communicable diseases risk factors in Brunei Darussalam. J Public Health (Berl).

[5] Philippine Heart Association-Council on Hypertension (2013). Philippine Heart Association-Council on Hypertension Report on Survey of Hypertension (Presyon 3): a report on prevalence of hypertension, awareness and treatment profile. Philipp J Cardiol.

[6] Castillo RR, Atilano AA, David-Ona DI (2019). May Measurement Month 2017: an analysis of blood pressure screening in the Philippines-South-East Asia and Australasia. Eur Heart J Suppl.

[7] Epidemiology and Disease Control Division. National Health Survey 2010. Singapore: Epidemiology and Disease Control Division; 2011. https://www.moh.gov.sg/‌docs/librariesprovider5/‌resources-statistics/‌reports/‌nhs2010---‌low-res.pdf

[8] Bureau of Policy and strategy (2007). Thailand health profile 2005–2006.

[9] Buranakitjaroen P, Wanthong S, Sukonthasarn A (2020). Asian management of hypertension: current status, home blood pressure, and specific concerns in Thailand. J Clin Hypertens (Greenwich).

[10] Sakboonyarat B, Rangsin R, Kantiwong A, Mungthin M (2019). Prevalence and associated factors of uncontrolled hypertension among hypertensive patients: a nation-wide survey in Thailand. BMC Res Notes.

[11] Son PT, Quang NN, Viet NL (2012). Prevalence, awareness, treatment and control of hypertension in Vietnam-results from a national survey. J Hum Hypertens.

[12] Beaney T, Schutte AE, Tomaszewski M (2018). May Measurement Month 2017: an analysis of blood pressure screening results worldwide [published correction appears in Lancet Glob Health. 2018 May 23;:]. Lancet Glob Health.

[13] Turana Y, Widyantoro B, Situmorang TD (2020). May Measurement Month 2018: an analysis of blood pressure screening results from Indonesia. Eur Heart J Suppl.

[14] Turana Y, Tengkawan J, Soenarta AA (2020). Asian management of hypertension: current status, home blood pressure, and specific concerns in Indonesia. J Clin Hypertens (Greenwich).

[15] Pengpid S, Vonglokham M, Kounnavong S, Sychareun V, Peltzer K (2019). The prevalence, awareness, treatment, and control of hypertension among adults: the first cross-sectional national population-based survey in Laos. Vasc Health Risk Manag.

[16] Vang C, Melanie C, Phoxay C, et al. Report on STEPS Survey on Non Communicable Diseases Rick Factors in Vientiane Capital city. 2010. https://www.who.int/ncds/surveillance/‌steps/2008_STEPS_Report_Laos.pdf

[17] Oum S, Prak PR, Khuon EM, et al. Prevalence of non-communicable disease risk factors in Cambodia. 2010. https://www.who.int/ncds/surveillance/steps/2010_STEPS_Report_‌Cambodia.pdf

[18] Gupta V, LoGerfo JP, Raingsey PP, Fitzpatrick AL (2013). The prevalence and associated factors for prehypertension and hypertension in Cambodia. Heart Asia.

[19] Bjertness MB, Htet AS, Meyer HE (2016). Prevalence and determinants of hypertension in Myanmar—a nationwide cross-sectional study. BMC Public Health.

[20] General Directorate of Statistics (GDS) and International Classification of Functioning, Disability and Health (ICF). 2016 Timor-Leste Demographic and Health Survey Key Findings. Rockville, Maryland, USA: GDS and ICF; 2018.

[21] Weber MA, Schiffrin EL, White WB (2014). Clinical practice guidelines for the management of hypertension in the community: a statement by the American Society of Hypertension and the International Society of Hypertension. J Clin Hypertens (Greenwich).

[22] Whelton PK, Carey RM, Aronow WS (2018). 2017 ACC/AHA/AAPA/ABC/ACPM/AGS/APhA/ASH/ASPC/NMA/PCNA Guideline for the Prevention, Detection, Evaluation, and Management of High Blood Pressure in Adults: Executive Summary: A Report of the American College of Cardiology/American Heart Association Task Force on Clinical Practice Guidelines [published correction appears in Hypertension. 2018 Jun;71(6):e136-e139] [published correction appears in Hypertension. 2018 Sep;72(3):e33]. Hypertension.

[23] Williams B, Mancia G, Spiering W (2018). 2018 ESC/ESH Guidelines for the management of arterial hypertension: The Task Force for the management of arterial hypertension of the European Society of Cardiology and the European Society of Hypertension: The Task Force for the management of arterial hypertension of the European Society of Cardiology and the European Society of Hypertension [published correction appears in J Hypertens. 2019 Jan;37(1):226]. J Hypertens.

[24] Lewington S, Clarke R, Qizilbash N, Peto R, Collins R; Prospective Studies Collaboration. Age-specific relevance of usual blood pressure to vascular mortality: a meta-analysis of individual data for one million adults in 61 prospective studies [published correction appears in Lancet. 2003 Mar 22;361(9362):1060]. Lancet. 2002;360(9349):1903–1913. 10.1016/s0140-6736(02)11911-8 12493255

[25] Wright JT, Williamson JD, SPRINT Research Group (2015). A Randomized trial of intensive versus standard blood-pressure control [published correction appears in N Engl J Med. 2017 Dec 21;377(25):2506]. N Engl J Med.

[26] Dzudie A, Rayner B, Ojji D (2018). Roadmap to achieve 25% hypertension control in Africa by 2025. Glob Heart.

[27] Messerli FH, Bangalore S (2018). The blood pressure landscape: schism among guidelines, confusion among physicians, and anxiety among patients. J Am Coll Cardiol.

[28] Al-Ansary LA, Tricco AC, Adi Y (2013). A systematic review of recent clinical practice guidelines on the diagnosis, assessment and management of hypertension. PLoS ONE.

[29] Alper BS, Price A, van Zuuren EJ (2019). Consistency of recommendations for evaluation and management of hypertension. JAMA Netw Open.

[30] Malaysian Society of Hypertension. Clinical Practice Guidelines: Management of Hypertension. 5th ed. 2018. http://www.acadmed.org.my/view_file.cfm?fileid=894

[31] Cardiac Society, Brunei Darussalam. Brunei Darussalam National Hypertension Guideline 2019. 2019. https://cardiacsociety.org.bn/wp-content/uploads/2019/10/National-Hypertension-Guidelines.pdf

[32] Ministry of Health, Singapore. Hypertension: MOH Clinical Practice Guidelines 1/2017. Singapore: Ministry of Health, Singapore; 2017. https://www.moh.gov.sg/docs/librariesprovider4/guidelines/cpg_hypertension-booklet---nov-2017.pdf

[33] Thai Hypertension Society. 2019 Thai Guidelines on The Treatment of Hypertension. 2019. http://www.thaihypertension.org/files/HT%20guideline%202019.with%20watermark.pdf

[34] Indonesian Society of Hypertension. Treatment of Hypertension Consensus 2019. Jakarta, Indonesia: Indonesian Society of Hypertension; 2019. http://upload.inash.or.id/cdn/File/Update%20konsensus%202019.pdf

[35] Vietnam National Heart Association. 2018 VNHA/VSH Guidelines on Diagnosis And Treatment of Arterial Hypertension In Adults. 2018. http://www.vnha.org.vn/

[36] Reboussin DM, Allen NB, Griswold ME, et al. Systematic Review for the 2017 ACC/AHA/AAPA/ABC/ACPM/AGS/APhA/ASH/ASPC/NMA/PCNA Guideline for the Prevention, Detection, Evaluation, and Management of High Blood Pressure in Adults: A Report of the American College of Cardiology/American Heart Association Task Force on Clinical Practice Guidelines [published correction appears in Hypertension. 2018 Jun;71(6):e145]. Hypertension. 2018;71(6):e116-e135. 10.1161/HYP.0000000000000067 29133355

[37] Ettehad D, Emdin CA, Kiran A (2016). Blood pressure lowering for prevention of cardiovascular disease and death: a systematic review and meta-analysis. Lancet.

[38] Thomopoulos C, Parati G, Zanchetti A (2016). Effects of blood pressure lowering treatment in hypertension: 8. Outcome reductions vs. discontinuations because of adverse drug events—meta-analyses of randomized trials. J Hypertens.

[39] Thomopoulos C, Parati G, Zanchetti A (2015). Effects of blood pressure-lowering on outcome incidence in hypertension: 5. Head-to-head comparisons of various classes of antihypertensive drugs—overview and meta-analyses. J Hypertens.

[40] Cruickshank JM (2017). The role of beta-blockers in the treatment of hypertension. Adv Exp Med Biol.

[41] Thomopoulos C, Bazoukis G, Tsioufis C, Mancia G (2020). Beta-blockers in hypertension: overview and meta-analysis of randomized outcome trials. J Hypertens.

[42] Guo X, Zhang X, Zheng L (2013). Prehypertension is not associated with all-cause mortality: a systematic review and meta-analysis of prospective studies. PLoS ONE.

[43] Guo X, Zhang X, Guo L (2013). Association between pre-hypertension and cardiovascular outcomes: a systematic review and meta-analysis of prospective studies. Curr Hypertens Rep.

[44] Huang Y, Wang S, Cai X (2013). Prehypertension and incidence of cardiovascular disease: a meta-analysis. BMC Med.

[45] Huang Y, Cai X, Zhang J (2014). Prehypertension and Incidence of ESRD: a systematic review and meta-analysis. Am J Kidney Dis.

[46] Huang Y, Cai X, Li Y (2014). Prehypertension and the risk of stroke: a meta-analysis. Neurology.

[47] Huang Y, Su L, Cai X (2014). Association of all-cause and cardiovascular mortality with prehypertension: a meta-analysis. Am Heart J.

[48] Huang Y, Cai X, Liu C (2015). Prehypertension and the risk of coronary heart disease in Asian and Western populations: a meta-analysis. J Am Heart Assoc.

[49] Lee M, Saver JL, Chang B, Chang KH, Hao Q, Ovbiagele B (2011). Presence of baseline prehypertension and risk of incident stroke: a meta-analysis. Neurology.

[50] Shen L, Ma H, Xiang MX, Wang JA (2013). Meta-analysis of cohort studies of baseline prehypertension and risk of coronary heart disease. Am J Cardiol.

[51] Wang S, Wu H, Zhang Q, Xu J, Fan Y (2013). Impact of baseline prehypertension on cardiovascular events and all-cause mortality in the general population: a meta-analysis of prospective cohort studies. Int J Cardiol.

[52] Brunström M, Carlberg B (2018). Association of blood pressure lowering with mortality and cardiovascular disease across blood pressure levels: a systematic review and meta-analysis. JAMA Internal Med.

[53] Kow CS, Hasan SS, Wong PS, Verma RK. Quality of clinical practice guidelines for the management of hypertension in six Southeast Asian countries. Clin Exp Hypertens. 2021. 10.1080/10641963.2021.1925683. 10.1080/10641963.2021.1925683 34092170

