# Supplementary material for: Concordance of recommendations across clinical practice guidelines for the management of hypertension in Southeast Asia with internationally reputable sources
Source: BMC Cardiovasc Disord. 2021 Jul 28;21:354. doi: 10.1186/s12872-021-02054-x (PMC8317337; doi:10.1186/s12872-021-02054-x)
Supplement: Supplementary file 1 — Additional file 1: Table S1. Detailed description of reference recommendations. Table S2. Concordance of reference recommendations generated from the 2017 ACC/AHA guideline. Table S3. Concordance of reference recommendations generated from the 2018 ESC/ESH guideline. Table S4. Concordance of reference recommendations generated from the 2017 ACC/AHA guideline after leave-one-out sensitivity analysis. Table S5. Concordance of reference recommendations generated from the 2018 ESC/ESH guideline after leave-one-out sensitivity analysis. [file 12872_2021_2054_MOESM1_ESM.docx]

**Table S1**: Detailed description of reference recommendations

| Code | Description of Reference Recommendation |
| --- | --- |
| ACC-1  ESC-1 | In all patients, blood pressure should be measured by following specific instructions on cuff size and body position of patients. |
| ACC-2  ESC-2 | In adults with suspected hypertension, diagnosis of hypertension using office blood pressure measurements is based on at least 2 measurements per office visit at from at least 2 office visits. |
| ACC-3 | In adults with suspected hypertension, diagnosis of hypertension using (non-automated) office blood pressure measurements is based on systolic blood pressure of ≥ 130 mm Hg and/or diastolic blood pressure of ≥ 80 mm Hg. |
| ESC-3 | In adults with suspected hypertension, diagnosis of hypertension using (non-automated) office blood pressure measurements is based on systolic blood pressure of ≥140 mm Hg and/or diastolic blood pressure of ≥90 mm Hg. |
| ACC-4 | In adults with suspected hypertension but without diagnostic uncertainty and blood pressure variability, ambulatory blood pressure monitoring is used to confirm the diagnosis of hypertension. |
| ACC-5 | In adults with suspected hypertension but without diagnostic uncertainty and blood pressure variability, home blood pressure monitoring is used to confirm the diagnosis of hypertension. |
| ACC-6  ESC-4 | In adults with suspected hypertension but with diagnostic uncertainty, ambulatory blood pressure monitoring is used to confirm the diagnosis of hypertension. |
| ESC-5 | In adults with suspected blood pressure variability, ambulatory blood pressure monitoring is used to confirm the diagnosis of hypertension. |
| ACC-7  ESC-6 | In adults with newly diagnosed hypertension, perform a baseline blood chemistry analysis (sodium, potassium, creatinine). |
| ACC-8  ESC-7 | In adults with newly diagnosed hypertension, perform a baseline fasting blood glucose level test. |
| ACC-9  ESC-8 | In adults with newly diagnosed hypertension, perform a baseline fasting lipid profile lest. |
| ACC-10  ESC-9 | In adults with newly diagnosed hypertension, perform a baseline dipstick urinalyis for the detection of blood and protein. |
| ACC-11  ESC-10 | In adults with newly diagnosed with hypertension, perform a baseline electrocardiography. |
| ACC-12  ESC-11 | In adults with newly diagnosed hypertension, perform a baseline serum hemoglobin or hematocrit level test. |
| ACC-13 | In adults with newly diagnosed hypertension, perform a baseline serum calcium level test. |
| ACC-14  ESC-12 | In adults with newly diagnosed hypertension, perform a baseline serum uric acid level test. |
| ESC-13 | In adults with newly diagnosed hypertension, perform baseline urine testing for albumin: creatinine ratio to quantify baseline albumin level. |
| ACC-15 | In adults with newly diagnosed hypertension, perform baseline urine testing for albumin to creatinine ratio instead of baseline 24-hour urine testing to quantify baseline albumin level. |
| ACC-16  ESC-14 | In adults with newly diagnosed hypertension, conduct targeted screening for potential underlying causes of secondary hypertension. |
| ACC-17  ESC-15 | In adults with newly diagnosed hypertension and with suspected structural heart disease, perform echocardiography. |
| ACC-18  ESC-16 | In adults with hypertension and concurrent overweight or obesity, provide counseling for weight loss. |
| ACC-19  ESC-17 | In adults with hypertension, provide counseling for dietary modifications. |
| ACC-20  ESC-18 | In adults with hypertension, provide counseling for regular physical activity. |
| ACC-21  ESC-19 | In adults with hypertension who smoke, counsel patients for smoking cessation. |
| ACC-22  ESC-20 | In adults with hypertension, provide counseling for salt or sodium restriction. |
| ACC-23  ESC-21 | In adults with hypertension and heavy alcohol consumption, counsel patients to moderate alcohol consumption. |
| ACC-24 | In adults age 18-60 years with hypertension but without diabetes, coronary artery disease, and chronic kidney disease, goal blood pressure of < 130/80 mm Hg is recommended. |
| ESC-22 | In adults age 18-60 years with hypertension but without diabetes, coronary artery disease, and chronic kidney disease, goal blood pressure of < 140/90 mm Hg is recommended. |
| ACC-25 | In adults age 60-80 years with hypertension but without diabetes, coronary artery disease, and chronic kidney disease, goal blood pressure of < 130/80 mm Hg is recommended. |
| ESC-23 | In adults age 60-80 years with hypertension but without diabetes, coronary artery disease, and chronic kidney disease, goal blood pressure of < 140/90 mm Hg is recommended. |
| ACC-26 | In adults age > 50 years with increased risk of cardiovascular disease, goal systolic blood pressure of <130 mm Hg is recommended. |
| ACC-27 | In adults age > 75-80 years with hypertension, goal blood pressure of < 130/80 mm Hg is recommended. |
| ESC-24 | In adults aged > 75-80 years with hypertension, goal blood pressure of < 140/90 mm Hg is recommended. |
| ACC-28 | In adults with hypertension and concurrent diabetes, goal blood pressure of < 130/80 mm Hg is recommended. |
| ESC-25 | In adults with hypertension and concurrent diabetes, goal blood pressure of < 140/80 mm Hg is recommended. |
| ACC-29 | In adults with hypertension and concurrent chronic kidney disease but without proteinuria and diabetes, goal blood pressure of < 130/80 mm Hg is recommended. |
| ESC-26 | In adults with hypertension and concurrent chronic kidney disease but without proteinuria and diabetes, goal blood pressure of < 140/90 mm Hg is recommended. |
| ACC-30  ESC-27 | In adults with hypertension and concurrent chronic kidney disease with proteinuria, goal blood pressure of < 130/80 mm Hg is recommended. |
| ACC-31 | In adults with hypertension and concurrent chronic kidney disease and diabetes, goal blood pressure of < 130/80 mm Hg is recommended. |
| ESC-28 | In adults with hypertension and concurrent chronic kidney disease and diabetes, goal blood pressure of < 140/90 mm Hg is recommended. |
| ACC-32  ESC-29 | In adults age < 60 years with hypertension but with no comorbidity requiring initial pharmacotherapy, thiazide diuretics are recommended as a therapeutic option for first-line therapy. |
| ACC-33  ESC-30 | In adults aged ≥ 60 years with hypertension but with no comorbidity requiring initial pharmacotherapy, thiazide diuretics are recommended as a therapeutic option for first-line therapy. |
| ACC-34  ESC-31 | In adults age < 60 years with hypertension but with no comorbidity requiring initial pharmacotherapy, ACE inhibitors are recommended as a therapeutic option for first-line therapy. |
| ACC-35  ESC-32 | In adults aged ≥ 60 years with hypertension but with no comorbidity requiring initial pharmacotherapy, ACE inhibitors are recommended as a therapeutic option for first-line therapy. |
| ACC-36  ESC-33 | In adults age < 60 years with hypertension but with no comorbidity requiring initial pharmacotherapy, ARBs are recommended as a therapeutic option for first-line therapy. |
| ACC-37  ESC-34 | In adults aged ≥ 60 years with hypertension but with no comorbidity requiring initial pharmacotherapy, ARBs are recommended as a therapeutic option for first-line therapy. |
| ACC-38  ESC-35 | In adults age < 60 years with hypertension but with no comorbidity requiring initial pharmacotherapy, CCBs are recommended as a therapeutic option for first-line therapy. |
| ACC-39  ESC-36 | In adults aged ≥ 60 years with hypertension but with no comorbidity requiring initial pharmacotherapy, CCBs are recommended as a therapeutic option for first-line therapy. |
| ACC-40 | In adults with hypertension but with no comorbidity requiring initial pharmacotherapy, beta-blockers are not recommended as a therapeutic option for first-line therapy. |
| ESC-37 | In adults with hypertension but with no comorbidity requiring initial pharmacotherapy, beta-blockers are recommended as a therapeutic option for first-line therapy. |
| ACC-41  ESC-38 | In adults with hypertension but with no comorbidity requiring initial pharmacotherapy, thiazide diuretics, ACE inhibitors, ARBs, and CCBs are preferred over beta-blockers as a therapeutic option for first-line therapy. |
| ACC-42  ESC-39 | In adults with hypertension and concurrent diabetes, ACE inhibitors or ARBs are recommended as a therapeutic option for first-line therapy. |
| ACC-43 | In adults with hypertension and concurrent diabetes, ACE inhibitors, ARBs, CCBs, or thiazide diuretics are recommended as the preferred option for first-line therapy. |
| ACC-44  ESC-40 | In adults with hypertension and concurrent chronic kidney disease, ACE inhibitors are recommended as a therapeutic option for first-line therapy. |
| ACC-45  ESC-41 | In adults with hypertension and concurrent chronic kidney disease, ARBs are recommended as equally preferred therapeutic option as ACE inhibitors for first-line therapy. |
| ACC-46 | In adults with hypertension and concurrent chronic kidney disease but without moderately increased albuminuria, thiazide diuretics or CCBs are recommended as equally preferred therapeutic option as ACE inhibitors or ARBs for first-line therapy. |
| ACC-47  ESC-42 | In adults with hypertension and concurrent chronic kidney disease with at least moderately increased albuminuria, ACE inhibitors or ARBs are recommended as the preferred therapeutic option for first-line therapy. |
| ACC-48 | In adults with hypertension and concurrent chronic kidney disease who are intolerant to ACE inhibitors, ARBs are recommended as a therapeutic option for first-line therapy. |
| ACC-49 | In adults with hypertension and concurrent chronic kidney disease who are intolerant to ACE inhibitors, ARBs are recommended as the preferred therapeutic option for first-line therapy. |
| ACC-50  ESC-43 | In adults with hypertension and concurrent coronary artery disease (i.e., ischemic heart disease) but without prior acute coronary syndrome, ACE inhibitors are recommended as a therapeutic option for first-line therapy. |
| ACC-51 | In adults with hypertension and prior acute coronary syndrome, ACE inhibitors are recommended as a therapeutic option for first-line therapy. |
| ACC-52  ESC-44 | In adults with hypertension and concurrent coronary artery disease (i.e., ischemic heart disease) but without prior acute coronary syndrome, ARBs are recommended as equally preferred therapeutic option as ACE inhibitors for first-line therapy. |
| ACC-53 | In adults with hypertension and prior acute coronary syndrome, ARBs are recommended as equally preferred therapeutic option as ACE inhibitors for first-line therapy. |
| ESC-45 | In adults with hypertension and concurrent coronary artery disease (i.e., ischemic heart disease) but without prior acute coronary syndrome, beta-blockers or CCBs are recommended as equally or more preferred therapeutic option than ACE inhibitors or ARBs for first-line therapy. |
| ESC-46 | In adults with hypertension and prior acute coronary syndrome, ACE inhibitors, ARBs, or beta-blockers are recommended as the preferred therapeutic option for first-line therapy. |
| ACC-54  ESC-47 | In adults with hypertension and concurrent coronary artery disease (i.e., ischemic heart disease) but without prior acute coronary syndrome who are intolerant to ACE inhibitors, ARBs are recommended as a therapeutic option for first-line therapy. |
| ACC-55 | In adults with hypertension and prior acute coronary syndrome who are intolerant to ACE inhibitors, ARBs are recommended as a therapeutic option for first-line therapy. |
| ESC-48 | In adults with hypertension and concurrent coronary artery disease (i.e., ischemic heart disease) but without prior acute coronary syndrome who are intolerant to ACE inhibitors, beta-blockers or CCBs are recommended as the preferred therapeutic option for first-line therapy. |
| ACC-56  ESC-49 | In adults with hypertension and recent acute coronary syndrome, beta-blockers are recommended as a therapeutic option for first-line therapy. |
| ACC-57  ESC-50 | In adults with hypertension and concurrent heart failure, ACE inhibitors are recommended as a therapeutic option for first-line therapy. |
| ACC-58  ESC-51 | In adults with hypertension and concurrent heart failure, beta-blockers are recommended as a therapeutic option for first-line therapy. |
| ACC-59 | In adults with hypertension and concurrent heart failure who are intolerant to ACE inhibitors, ARBs are recommended as a therapeutic option for first-line therapy. |

**Table S2**: Concordance of reference recommendations generated from the 2017 ACC/AHA guideline

| Code | Population | Intervention | Comparator | MAL | BRN | SGP | THA | IDN | VNM | Concordance of recommendation | Concordance of recommendation after exclusion of “insufficient” | Does excluding 'Insufficient' make a difference in concordance of recommendation? |
| --- | --- | --- | --- | --- | --- | --- | --- | --- | --- | --- | --- | --- |
| ACC-1 | **all patients** | **blood pressure measurement with specific instructions on cuff size and body position of patients** | **blood pressure measurement without specific instructions** | √ | √ | √ | √ | √ | INS | Yes | Yes | No |
| ACC-2 | **adults with suspected hypertension** | **diagnosis based on at least 2 blood pressure measurements per office visit from at least 2 office visits** | **diagnosis based on single blood pressure measurement and/or single office visit** | √ | √ | √ | √ | √ | √ | Yes | Yes | No |
| ACC-3 | **adults with suspected hypertension** | **diagnosis based on office (non-automated) systolic blood pressure measurement of > 130 mm Hg or diastolic blood pressure measurement of > 80 mm Hg** | **diagnosis based on thresholds other than systolic blood pressure measurement of > 130 mm Hg or diastolic blood pressure measurement of > 80 mm Hg** | X | X | X | X | X | X | No | No | No |
| ACC-4 | **adults with suspected hypertension but without diagnostic uncertainty and blood pressure variability** | **ambulatory blood pressure monitoring to confirm the diagnosis of hypertension** | **diagnosis based on office blood pressure measurements alone** | X | X | X | √ | √ | √ | No | No | No |
| ACC-5 | **adults with suspected hypertension but without diagnostic uncertainty and blood pressure variability** | **home blood pressure monitoring to confirm the diagnosis of hypertension** | **diagnosis based on office blood pressure measurements alone** | INS | X | X | √ | √ | √ | No | No | No |
| ACC-6 | **adults with suspected hypertension but with diagnostic uncertainty** | **performance of ambulatory blood pressure monitoring to confirm the diagnosis of hypertension** | **performance of ambulatory blood pressure monitoring to confirm the diagnosis of hypertension** | √ | √ | √ | INS | INS | INS | No | Yes | Yes |
| ACC-7 | **adults newly diagnosed with hypertension** | **performance of baseline blood chemistry (sodium, potassium, creatinine)** | **no performance of baseline blood chemistry** | √ | INS | √ | √ | X | √ | No | No | No |
| ACC-8 | **adults newly diagnosed with hypertension** | **performance of baseline fasting blood glucose level** | **no performance of baseline fasting blood glucose level test** | √ | √ | √ | √ | X | √ | No | No | No |
| ACC-9 | **adults newly diagnosed with hypertension** | **performance of baseline fasting lipid profile test** | **no performance of baseline fasting lipid profile test** | √ | √ | √ | √ | X | √ | No | No | No |
| ACC-10 | **adults newly diagnosed with hypertension** | **performance of baseline dipstick urinalyis for the detection of blood and protein** | **no performance of baseline dipstick urinalyis** | √ | √ | √ | √ | √ | √ | Yes | Yes | No |
| ACC-11 | **adults newly diagnosed with hypertension** | **performance of baseline electrocardiography** | **no performance of baseline electrocardiography** | √ | √ | √ | √ | √ | √ | Yes | Yes | No |
| ACC-12 | **adults newly diagnosed with hypertension** | **performance of baseline serum hemoglobin or hematocrit level test** | **no performance of baseline serum hemoglobin or hematocrit level test** | √ | √ | X | √ | X | √ | No | No | No |
| ACC-13 | **adults newly diagnosed with hypertension** | **performance of baseline serum calcium level test** | **no performance of baseline serum calcium level test** | √ | INS | √ | X | X | X | No | No | No |
| ACC-14 | **adults newly diagnosed with hypertension** | **performance of baseline serum uric acid level test** | **no performance of baseline serum uric acid level test** | √ | √ | X | √ | X | √ | No | No | No |
| ACC-15 | **adults newly diagnosed with hypertension** | **performance of urine testing for albumin:creatinine ratio to quantify baseline albumin level** | **performance of 24-h to quantify baseline albumin level** | DIF | INS | INS | INS | DIF | √ | No | Yes | Yes |
| ACC-16 | **adults newly diagnosed with hypertension** | **targeted screening for potential underlying causes of secondary hypertension** | **no screening for potential underlying causes of secondary hypertension** | √ | √ | √ | √ | √ | √ | Yes | Yes | No |
| ACC-17 | **adults newly diagnosed with hypertension and with suspected structural heart disease** | **Performance of baseline echocardiography** | **no performance of baseline echocardiography** | OOS | OOS | √ | √ | √ | INS | Yes | Yes | No |
| ACC-18 | **adults with hypertension and concurrent overweight/obesity** | **counseling for weight loss** | **no counseling for weight loss** | √ | √ | √ | √ | √ | √ | Yes | Yes | No |
| ACC-19 | **adults with hypertension** | **counseling for dietary modifications** | **no counseling for dietary modifications** | √ | √ | √ | √ | √ | √ | Yes | Yes | No |
| ACC-20 | **adults with hypertension** | **counseling for regular physical activity** | **no counseling for regular physical activity** | √ | √ | √ | √ | √ | √ | Yes | Yes | No |
| ACC-21 | **adults with hypertension who smoke** | **counseling for smoking cessation** | **no counseling for smoking cessation** | √ | √ | √ | √ | √ | √ | Yes | Yes | No |
| ACC-22 | **adults with hypertension** | **counseling for salt/sodium restriction** | **no counseling for salt/sodium restriction** | √ | √ | √ | √ | √ | √ | Yes | Yes | No |
| ACC-23 | **adults with hypertension and heavy alcohol consumption** | **counseling to moderate alcohol consumption** | **no counseling on alcohol consumption** | √ | √ | √ | √ | √ | √ | Yes | Yes | No |
| ACC-24 | **adults age 18-60 years with hypertension but without diabetes, coronary artery disease, and chronic kidney disease** | **goal blood pressure of < 130/80 mm Hg** | **other goal blood pressure** | X | X | X | √ | √ | √ | No | No | No |
| ACC-25 | **adults age 60-80 years with hypertension but without diabetes, coronary artery disease, and chronic kidney disease** | **goal blood pressure of < 130/80 mm Hg** | **other goal blood pressure** | X | X | X | X | X | X | No | No | No |
| ACC-26 | **adults age > 50 years with increased risk of cardiovascular disease** | **goal systolic blood pressure of < 130 mm Hg** | **other goal systolic blood pressure** | X | INS | X | INS | INS | INS | No | No | No |
| ACC-27 | **adults age > 75-80 years with hypertension** | **goal blood pressure of < 130/80 mm Hg** | **other goal blood pressure** | X | X | X | X | X | X | No | No | No |
| ACC-28 | **adults with hypertension and concurrent diabetes** | **goal blood pressure of < 130/80 mm Hg** | **other goal blood pressure** | X | √ | X | √ | √ | √ | No | No | No |
| ACC-29 | **adults with hypertension and concurrent chronic kidney disease but without proteinuria and diabetes** | **goal blood pressure of < 130/80 mm Hg** | **other goal blood pressure** | X | X | X | √ | X | X | No | No | No |
| ACC-30 | **adults with hypertension and concurrent chronic kidney disease with proteinuria** | **goal blood pressure of < 130/80 mm Hg** | **other goal blood pressure** | √ | √ | √ | √ | X | X | No | No | No |
| ACC-31 | **adults with hypertension and chronic kidney disease and diabetes** | **goal blood pressure of < 130/80 mm Hg** | **other goal blood pressure** | X | X | X | √ | X | X | No | No | No |
| ACC-32 | **adults age < 60 years with hypertension but with no**  **comorbidity requiring initial pharmacotherapy** | **thiazide diuretics as an option for first-line therapy** | **not recommending thiazide diuretics as an option** | √ | X | √ | √ | √ | √ | No | No | No |
| ACC-33 | **adults age ≥ 60 years with hypertension but with no**  **comorbidity requiring initial pharmacotherapy** | **thiazide diuretics as an option for first-line therapy** | **not recommending thiazide diuretics as an option** | √ | X | √ | √ | √ | √ | No | No | No |
| ACC-34 | **adults age < 60 years with hypertension but with no**  **comorbidity requiring initial pharmacotherapy** | **ACE inhibitors as an option for first-line therapy** | **not recommending ACE inhibitors as an option for first-line therapy** | √ | √ | √ | √ | √ | √ | Yes | Yes | No |
| ACC-35 | **adults age ≥ 60 years with hypertension but with no**  **comorbidity requiring initial pharmacotherapy** | **ACE inhibitors as an option for first-line therapy** | **not recommending ACE inhibitor as an option for first-line therapy** | √ | √ | √ | √ | √ | √ | Yes | Yes | No |
| ACC-36 | **adults age < 60 years with hypertension but with no**  **comorbidity requiring initial pharmacotherapy** | **ARBs as an option for first-line therapy** | **not recommending ARBs as an option for first-line therapy** | √ | √ | √ | √ | √ | √ | Yes | Yes | No |
| ACC-37 | **adults age ≥ 60 years with hypertension but with no**  **comorbidity requiring initial pharmacotherapy** | **ARBs as an option for first-line therapy** | **not recommending ARBs as an option for first-line therapy** | √ | √ | √ | √ | √ | √ | Yes | Yes | No |
| ACC-38 | **adults age < 60 years with hypertension but with no**  **comorbidity requiring initial pharmacotherapy** | **calcium channel blockers as an option for first-line therapy** | **not recommending calcium channel blockers as an option for first-line therapy** | √ | √ | √ | √ | √ | √ | Yes | Yes | No |
| ACC-39 | **adults age ≥ 60 years with hypertension but with no**  **comorbidity requiring initial pharmacotherapy** | **calcium channel blockers as an option for first-line therapy** | **not recommending calcium channel blockers as an option for first-line therapy** | √ | √ | √ | √ | √ | √ | Yes | Yes | No |
| ACC-40 | **adults with hypertension but with no**  **comorbidity requiring initial pharmacotherapy** | **not recommending beta-blockers as an option for first-line therapy** | **beta-blocker as an option for first-line therapy** | X | √ | X | X | √ | √ | No | No | No |
| ACC-41 | **adults with hypertension but with no**  **comorbidity requiring initial pharmacotherapy** | **thiazide diuretics, ACE inhibitors, ARBs, or calcium channel blockers are preferred over beta-blockers as an option for first-line therapy** | **beta blockers as equally preferred option as thiazide diuretics, ACE inhibitors, ARBs, and calcium channel blockers for first-line therapy** | X | √ | X | X | √ | √ | No | No | No |
| ACC-42 | **adults with hypertension and concurrent diabetes** | **ACE inhibitors or ARBs as an option for first-line therapy** | **not recommending ACE inhibitors or ARBs as an option for first-line therapy** | √ | √ | √ | √ | √ | √ | Yes | Yes | No |
| ACC-43 | **adults with hypertension and concurrent diabetes** | **ACE inhibitors, ARBs, calcium channel blockers, or thiazide diuretics as the preferred option for first-line therapy** | **other drug therapies are preferred over ACE inhibitors, ARBs, calcium channel blockers, or thiazide diuretics as an option for first-line therapy** | DIF | DIF | DIF | DIF | DIF | √ | Yes | Yes | No |
| ACC-44 | **adults with hypertension and concurrent chronic kidney disease** | **ACE inhibitors as an option for first-line therapy** | **not recommending ACE inhibitors as an option for first-line therapy** | INS | √ | √ | √ | √ | √ | Yes | Yes | No |
| ACC-45 | **adults with hypertension and concurrent chronic kidney disease** | **ARBs as equally preferred option as ACE inhibitors for first-line therapy** | **ACE inhibitors are preferred over ARBs as an option for first-line therapy** | INS | √ | √ | √ | √ | √ | Yes | Yes | No |
| ACC-46 | **adults with hypertension and concurrent chronic kidney disease but without moderately increased albuminuria** | **thiazide diuretics or calcium channel blockers as equally preferred option as ACE inhibitors or ARBs for first-line therapy** | **ACE inhibitors or ARBs are preferred over thiazide diuretics or calcium channel blockers as an option for first-line therapy** | INS | √ | X | √ | √ | √ | No | No | No |
| ACC-47 | **adults with hypertension and concurrent chronic kidney disease with at least moderately increased albuminuria** | **ACE inhibitors or ARBs as the preferred option for first-line therapy** | **drug therapies other than ACE inhibitors or ARBs as equally or more preferred option for first-line therapy** | √ | √ | √ | √ | √ | √ | Yes | Yes | No |
| ACC-48 | **adults with hypertension and concurrent chronic kidney disease intolerant to ACE inhibitors** | **ARBs as an option for first-line therapy** | **not recommending ARBs as an option for first-line therapy** | INS | INS | INS | INS | INS | INS | No | N/A | Yes |
| ACC-49 | **adults with hypertension and concurrent chronic kidney disease intolerant to ACE inhibitors** | **ARBs as the preferred option for first-line therapy** | **drug therapies other than ARBs as equally or more preferred option for first-line therapy** | INS | INS | INS | INS | INS | INS | No | N/A | Yes |
| ACC-50 | **adults with hypertension and concurrent coronary artery disease (ischemic heart disease) but without prior acute coronary syndrome** | **ACE inhibitors as an option for first-line therapy** | **not recommending ACE inhibitors as an option for first-line therapy** | INS | √ | INS | √ | √ | √ | Yes | Yes | No |
| ACC-51 | **adults with hypertension and prior acute coronary syndrome** | **ACE inhibitors as an option for first-line therapy** | **not recommending ACE inhibitors as an option for first-line therapy** | √ | √ | √ | √ | √ | √ | Yes | Yes | No |
| ACC-52 | **adults with hypertension and concurrent coronary artery disease (ischemic heart disease) but without prior acute coronary syndrome** | **ARBs as equally preferred option as ACE inhibitors for first-line therapy** | **ACE inhibitors are preferred over ARBs as an option for first-line therapy** | INS | √ | INS | √ | √ | √ | Yes | Yes | No |
| ACC-53 | **adults with hypertension and prior acute coronary syndrome** | **ARBs as equally preferred option as ACE inhibitors for first-line therapy** | **ACE inhibitors are preferred over ARBs as an option for first-line therapy** | √ | √ | √ | √ | √ | √ | Yes | Yes | No |
| ACC-54 | **adults with hypertension and concurrent coronary artery disease (ischemic heart disease) but without prior acute coronary syndrome intolerant to ACE inhibitor** | **ARBs as an option for first-line therapy** | **not recommending ARBs as an option for first-line therapy** | INS | INS | INS | INS | INS | INS | No | N/A | Yes |
| ACC-55 | **adults with hypertension and prior acute coronary syndrome intolerant to ACE inhibitor** | **ARBs as an option for first-line therapy** | **not recommending ARBs as an option for first-line therapy** | INS | INS | INS | INS | INS | INS | No | N/A | Yes |
| ACC-56 | **adults with hypertension and recent acute coronary syndrome** | **beta-blockers as an option for first-line therapy** | **not recommending beta-blockers as an option for first-line therapy** | √ | √ | √ | √ | √ | √ | Yes | Yes | No |
| ACC-57 | **adults with hypertension and concurrent heart failure** | **ACE inhibitors as an option for first-line therapy** | **not recommending ACE inhibitors as an option for first-line therapy** | √ | √ | √ | √ | √ | √ | Yes | Yes | No |
| ACC-58 | **adults with hypertension and concurrent heart failure** | **beta-blockers as an option for first-line therapy** | **not recommending beta-blockers as an option for first-line therapy** | √ | √ | √ | √ | √ | √ | Yes | Yes | No |
| ACC-59 | **adults with hypertension and concurrent heart failure intolerant to ACE inhibitors** | **ARBs as an option for first-line therapy** | **not recommending ARBs as an option for first-line therapy** | √ | INS | INS | INS | INS | INS | No | Yes | Yes |

**Table S3**: Concordance of reference recommendations generated from the 2018 ESC/ESH guideline

| Code | Population | Intervention | Comparator | MAL | BRN | SGP | THA | IDN | VNM | Concordance of recommendation | Concordance of recommendation after exclusion of “insufficient” | Does excluding 'Insufficient' make a difference in concordance of recommendation? |
| --- | --- | --- | --- | --- | --- | --- | --- | --- | --- | --- | --- | --- |
| ESC-1 | **all patients** | **blood pressure measurement with specific instructions on cuff size and body position of patients** | **blood pressure measurement without specific instructions** | √ | √ | √ | √ | √ | INS | Yes | Yes | No |
| ESC-2 | **adults with suspected hypertension** | **diagnosis based on at least 2 blood pressure measurements per office visit from at least 2 office visits** | **diagnosis based on single blood pressure measurement and/or single office visit** | √ | √ | √ | √ | √ | √ | Yes | Yes | No |
| ESC-3 | **adults with suspected hypertension** | **diagnosis based on office (non-automated) systolic blood pressure measurement of > 140 mm Hg or diastolic blood pressure measurement of > 90 mm Hg** | **diagnosis based on thresholds other than systolic blood pressure measurement of > 140 mm Hg or diastolic blood pressure measurement of > 90 mm Hg** | √ | √ | √ | √ | √ | √ | Yes | No | No |
| ESC-4 | **adults with suspected hypertension but with diagnostic uncertainty** | **performance of ambulatory blood pressure monitoring to confirm the diagnosis of hypertension** | **performance of ambulatory blood pressure monitoring to confirm the diagnosis of hypertension** | √ | √ | √ | INS | INS | INS | No | Yes | **Yes** |
| ESC-5 | **adults with suspected blood pressure variability** | **performance of ambulatory blood pressure monitoring to confirm the diagnosis of hypertension** | **performance of ambulatory blood pressure monitoring to confirm the diagnosis of hypertension** | √ | INS | √ | INS | √ | √ | Yes | No | No |
| ESC-6 | **adults newly diagnosed with hypertension** | **performance of baseline blood chemistry (sodium, potassium, creatinine)** | **no performance of baseline blood chemistry** | √ | INS | √ | √ | X | √ | No | **Yes** | No |
| ESC-7 | **adults newly diagnosed with hypertension** | **performance of baseline fasting blood glucose level** | **no performance of baseline fasting blood glucose level test** | √ | √ | √ | √ | X | √ | No | No | No |
| ESC-8 | **adults newly diagnosed with hypertension** | **performance of baseline fasting lipid profile test** | **no performance of baseline fasting lipid profile test** | √ | √ | √ | √ | X | √ | No | No | No |
| ESC-9 | **adults newly diagnosed with hypertension** | **performance of baseline dipstick urinalyis for the detection of blood and protein** | **no performance of baseline dipstick urinalyis** | √ | √ | √ | √ | √ | √ | Yes | No | No |
| ESC-10 | **adults newly diagnosed with hypertension** | **performance of baseline electrocardiography** | **no performance of baseline electrocardiography** | √ | √ | √ | √ | √ | √ | Yes | Yes | No |
| ESC-11 | **adults newly diagnosed with hypertension** | **performance of baseline serum hemoglobin or hematocrit level test** | **no performance of baseline serum hemoglobin or hematocrit level test** | √ | √ | X | √ | X | √ | No | Yes | No |
| ESC-12 | **adults newly diagnosed with hypertension** | **performance of baseline serum uric acid level test** | **no performance of baseline serum uric acid level test** | √ | √ | X | √ | X | √ | No | No | No |
| ESC-13 | **adults newly diagnosed with hypertension** | **performance of urine testing for albumin:creatinine ratio to quantify baseline albumin level** | **no performance of urine testing to quantify baseline albumin level** | X | √ | INS | INS | X | √ | No | No | No |
| ESC-14 | **adults newly diagnosed with hypertension** | **targeted screening for potential underlying causes of secondary hypertension** | **no screening for potential underlying causes of secondary hypertension** | √ | √ | √ | √ | √ | √ | Yes | No | No |
| ESC-15 | **adults newly diagnosed with hypertension and with suspected structural heart disease** | **Performance of baseline echocardiography** | **no performance of baseline echocardiography** | OOS | OOS | √ | √ | √ | INS | Yes | **Yes** | No |
| ESC-16 | **adults with hypertension and concurrent overweight/obesity** | **counseling for weight loss** | **no counseling for weight loss** | √ | √ | √ | √ | √ | √ | Yes | Yes | No |
| ESC-17 | **adults with hypertension** | **counseling for dietary modifications** | **no counseling for dietary modifications** | √ | √ | √ | √ | √ | √ | Yes | Yes | No |
| ESC-18 | **adults with hypertension** | **counseling for regular physical activity** | **no counseling for regular physical activity** | √ | √ | √ | √ | √ | √ | Yes | Yes | No |
| ESC-19 | **adults with hypertension who smoke** | **counseling for smoking cessation** | **no counseling for smoking cessation** | √ | √ | √ | √ | √ | √ | Yes | Yes | No |
| ESC-20 | **adults with hypertension** | **counseling for salt/sodium restriction** | **no counseling for salt/sodium restriction** | √ | √ | √ | √ | √ | √ | Yes | Yes | No |
| ESC-21 | **adults with hypertension and heavy alcohol consumption** | **counseling to moderate alcohol consumption** | **no counseling on alcohol consumption** | √ | √ | √ | √ | √ | √ | Yes | Yes | No |
| ESC-22 | **adults age 18-60 years with hypertension but without diabetes, coronary artery disease, and chronic kidney disease** | **goal blood pressure of < 140/90 mm Hg** | **other goal blood pressure** | √ | √ | √ | X | X | X | No | Yes | No |
| ESC-23 | **adults age 60-80 years with hypertension but without diabetes, coronary artery disease, and chronic kidney disease** | **goal blood pressure of < 140/90 mm Hg** | **other goal blood pressure** | √ | √ | √ | √ | X | X | No | Yes | No |
| ESC-24 | **adults age > 75-80 years with hypertension** | **goal blood pressure of < 140/90 mm Hg** | **other goal blood pressure** | X | √ | X | √ | X | X | No | No | No |
| ESC-25 | **adults with hypertension and concurrent diabetes** | **goal blood pressure of < 140/90 mm Hg** | **other goal blood pressure** | √ | X | √ | X | X | X | No | No | No |
| ESC-26 | **adults with hypertension and concurrent chronic kidney disease but without proteinuria and diabetes** | **goal blood pressure of < 140/90 mm Hg** | **other goal blood pressure** | √ | √ | √ | X | X | X | No | No | No |
| ESC-27 | **adults with hypertension and concurrent chronic kidney disease with proteinuria** | **goal blood pressure of < 130/80 mm Hg** | **other goal blood pressure** | √ | √ | √ | √ | X | X | No | No | No |
| ESC-28 | **adults with hypertension and chronic kidney disease and diabetes** | **goal blood pressure of < 140/90 mm Hg** | **other goal blood pressure** | X | √ | X | X | X | X | No | No | No |
| ESC-29 | **adults age < 60 years with hypertension but with no**  **comorbidity requiring initial pharmacotherapy** | **thiazide diuretics as an option for first-line therapy** | **not recommending thiazide diuretics as an option** | √ | X | √ | √ | √ | √ | No | No | No |
| ESC-30 | **adults age ≥ 60 years with hypertension but with no**  **comorbidity requiring initial pharmacotherapy** | **thiazide diuretics as an option for first-line therapy** | **not recommending thiazide diuretics as an option** | √ | X | √ | √ | √ | √ | No | No | No |
| ESC-31 | **adults age < 60 years with hypertension but with no**  **comorbidity requiring initial pharmacotherapy** | **ACE inhibitors as an option for first-line therapy** | **not recommending ACE inhibitors as an option for first-line therapy** | √ | √ | √ | √ | √ | √ | Yes | No | No |
| ESC-32 | **adults age ≥ 60 years with hypertension but with no**  **comorbidity requiring initial pharmacotherapy** | **ACE inhibitors as an option for first-line therapy** | **not recommending ACE inhibitor as an option for first-line therapy** | √ | √ | √ | √ | √ | √ | Yes | No | No |
| ESC-33 | **adults age < 60 years with hypertension but with no**  **comorbidity requiring initial pharmacotherapy** | **ARBs as an option for first-line therapy** | **not recommending ARBs as an option for first-line therapy** | √ | √ | √ | √ | √ | √ | Yes | No | No |
| ESC-34 | **adults age ≥ 60 years with hypertension but with no**  **comorbidity requiring initial pharmacotherapy** | **ARBs as an option for first-line therapy** | **not recommending ARBs as an option for first-line therapy** | √ | √ | √ | √ | √ | √ | Yes | Yes | No |
| ESC-35 | **adults age < 60 years with hypertension but with no**  **comorbidity requiring initial pharmacotherapy** | **calcium channel blockers as an option for first-line therapy** | **not recommending calcium channel blockers as an option for first-line therapy** | √ | √ | √ | √ | √ | √ | Yes | Yes | No |
| ESC-36 | **adults age ≥ 60 years with hypertension but with no**  **comorbidity requiring initial pharmacotherapy** | **calcium channel blockers as an option for first-line therapy** | **not recommending calcium channel blockers as an option for first-line therapy** | √ | √ | √ | √ | √ | √ | Yes | Yes | No |
| ESC-37 | **adults with hypertension but with no comorbidity requiring initial pharmacotherapy** | **beta-blockers as an option for first-line therapy** | **not recommending beta-blockers as an option for first-line therapy** | √ | X | √ | √ | X | X | No | Yes | No |
| ESC-38 | **adults with hypertension but with no**  **comorbidity requiring initial pharmacotherapy** | **thiazide diuretics, ACE inhibitors, ARBs, or calcium channel blockers are preferred over beta-blockers as an option for first-line therapy** | **beta blockers as equally preferred option as thiazide diuretics, ACE inhibitors, ARBs, and calcium channel blockers for first-line therapy** | X | √ | X | X | √ | √ | No | Yes | No |
| ESC-39 | **adults with hypertension and concurrent diabetes** | **ACE inhibitors or ARBs as an option for first-line therapy** | **not recommending ACE inhibitors or ARBs as an option for first-line therapy** | √ | √ | √ | √ | √ | √ | Yes | Yes | No |
| ESC-40 | **adults with hypertension and concurrent chronic kidney disease** | **ACE inhibitors as an option for first-line therapy** | **not recommending ACE inhibitors as an option for first-line therapy** | INS | √ | √ | √ | √ | √ | Yes | No | No |
| ESC-41 | **adults with hypertension and concurrent chronic kidney disease** | **ARBs as equally preferred option as ACE inhibitors for first-line therapy** | **ACE inhibitors are preferred over ARBs as an option for first-line therapy** | INS | √ | √ | √ | √ | √ | Yes | No | No |
| ESC-42 | **adults with hypertension and concurrent chronic kidney disease with at least moderately increased albuminuria** | **ACE inhibitors or ARBs as the preferred option for first-line therapy** | **drug therapies other than ACE inhibitors or ARBs as equally or more preferred option for first-line therapy** | √ | √ | √ | √ | √ | √ | Yes | Yes | No |
| ESC-43 | **adults with hypertension and concurrent coronary artery disease (ischemic heart disease) but without prior acute coronary syndrome** | **ACE inhibitors as an option for first-line therapy** | **not recommending ACE inhibitors as an option for first-line therapy** | INS | √ | INS | √ | √ | √ | Yes | Yes | No |
| ESC-44 | **adults with hypertension and concurrent coronary artery disease (ischemic heart disease) but without prior acute coronary syndrome** | **ARBs as equally preferred option as ACE inhibitors for first-line therapy** | **ACE inhibitors are preferred over ARBs as an option for first-line therapy** | INS | √ | INS | √ | √ | √ | Yes | Yes | No |
| ESC-45 | **adults with hypertension and concurrent coronary artery disease (ischemic heart disease) but without prior acute coronary syndrome** | **beta-blockers or calcium channel blockers as equally or more preferred option than ACE inhibitors or ARBs for first-line therapy** | **ACE inhibitors or ARBs are preferred over beta-blockers or calcium channel blockers as an option for first-line therapy** | INS | √ | √ | √ | √ | √ | Yes | Yes | No |
| ESC-46 | **adults with hypertension and prior acute coronary syndrome** | **beta-blockers, ACE inhibitors, or ARBs as the preferred option for first-line therapy** | **other drug therapies as equally or more preferred option than beta-blockers, ACE inhibitors, or ARBs for first-line therapy** | √ | √ | √ | √ | √ | √ | Yes | No | No |
| ESC-47 | **adults with hypertension and concurrent coronary artery disease (ischemic heart disease) but without prior acute coronary syndrome intolerant to ACE inhibitor** | **ARBs as an option for first-line therapy** | **not recommending ARBs as an option for first-line therapy** | INS | INS | INS | INS | INS | INS | No | **N/A** | **Yes** |
| ESC-48 | **adults with hypertension and concurrent coronary artery disease (ischemic heart disease) but without prior acute coronary syndrome intolerant to ACE inhibitor** | **beta-blockers or calcium channel blockers as the preferred option for first-line therapy** | **ARBs as equally or more preferred option than beta-blockers or calcium channel blockers for first-line therapy** | INS | INS | INS | INS | INS | INS | No | **N/A** | **Yes** |
| ESC-49 | **adults with hypertension and recent acute coronary syndrome** | **beta-blockers as an option for first-line therapy** | **not recommending beta-blockers as an option for first-line therapy** | √ | √ | √ | √ | √ | √ | Yes | Yes | No |
| ESC-50 | **adults with hypertension and concurrent heart failure** | **ACE inhibitors as an option for first-line therapy** | **not recommending ACE inhibitors as an option for first-line therapy** | √ | √ | √ | √ | √ | √ | Yes | Yes | No |
| ESC-51 | **adults with hypertension and concurrent heart failure** | **beta-blockers as an option for first-line therapy** | **not recommending beta-blockers as an option for first-line therapy** | √ | √ | √ | √ | √ | √ | Yes | Yes | No |

**Table S4**: Concordance of reference recommendations generated from the 2017 ACC/AHA guideline after leave-one-out sensitivity analysis

| **Code** | **Concordance of recommendation before leave-one-out sensitivity analysis** | **Concordance of recommendation after exclusion of ‘MAL’** | **Does excluding 'MAL' make a difference in concordance of recommendation?** | **Concordance of recommendation after exclusion of ‘BRN’** | **Does excluding 'BRN' make a difference in concordance of recommendation?** | **Concordance of recommendation after exclusion of ‘SGP’** | **Does excluding 'SGP' make a difference in concordance of recommendation?** | **Concordance of recommendation after exclusion of ‘THA’** | **Does excluding 'THA' make a difference in concordance of recommendation?** | **Concordance of recommendation after exclusion of ‘IDN’** | **Does excluding 'IDN' make a difference in concordance of recommendation?** | **Concordance of recommendation after exclusion of ‘VNM’** | **Does excluding 'VNM' make a difference in concordance of recommendation?** |
| --- | --- | --- | --- | --- | --- | --- | --- | --- | --- | --- | --- | --- | --- |
|  | Yes | Yes | No | Yes | No | Yes | No | Yes | No | Yes | No | Yes | No |
|  | Yes | Yes | No | Yes | No | Yes | No | Yes | No | Yes | No | Yes | No |
|  | No | No | No | No | No | No | No | No | No | No | No | No | No |
|  | No | No | No | No | No | No | No | No | No | No | No | No | No |
|  | No | No | No | No | No | No | No | No | No | No | No | No | No |
|  | No | No | No | No | No | No | No | Yes | Yes | Yes | Yes | Yes | Yes |
|  | No | No | No | No | No | No | No | No | No | No | No | No | No |
|  | No | No | No | No | No | No | No | No | No | Yes | Yes | No | No |
|  | No | No | No | No | No | No | No | No | No | Yes | Yes | No | No |
|  | Yes | Yes | No | Yes | No | Yes | No | Yes | No | Yes | No | Yes | No |
|  | Yes | Yes | No | Yes | No | Yes | No | Yes | No | Yes | No | Yes | No |
|  | No | No | No | No | No | No | No | No | No | No | No | No | No |
|  | No | No | No | No | No | No | No | No | No | No | No | No | No |
|  | No | No | No | No | No | No | No | No | No | No | No | No | No |
|  | No | No | No | No | No | No | No | No | No | No | No | No | No |
|  | Yes | Yes | No | Yes | No | Yes | No | Yes | No | Yes | No | Yes | No |
|  | Yes | Yes | No | Yes | No | Yes | No | Yes | No | Yes | No | Yes | No |
|  | Yes | Yes | No | Yes | No | Yes | No | Yes | No | Yes | No | Yes | No |
|  | Yes | Yes | No | Yes | No | Yes | No | Yes | No | Yes | No | Yes | No |
|  | Yes | Yes | No | Yes | No | Yes | No | Yes | No | Yes | No | Yes | No |
|  | Yes | Yes | No | Yes | No | Yes | No | Yes | No | Yes | No | Yes | No |
|  | Yes | Yes | No | Yes | No | Yes | No | Yes | No | Yes | No | Yes | No |
|  | Yes | Yes | No | Yes | No | Yes | No | Yes | No | Yes | No | Yes | No |
|  | No | No | No | No | No | No | No | No | No | No | No | No | No |
|  | No | No | No | No | No | No | No | No | No | No | No | No | No |
|  | No | No | No | No | No | No | No | No | No | No | No | No | No |
|  | No | No | No | No | No | No | No | No | No | No | No | No | No |
|  | No | No | No | No | No | No | No | No | No | No | No | No | No |
|  | No | No | No | No | No | No | No | No | No | No | No | No | No |
|  | No | No | No | No | No | No | No | No | No | No | No | No | No |
|  | No | No | No | No | No | No | No | No | No | No | No | No | No |
|  | No | No | No | Yes | Yes | No | No | No | No | No | No | No | No |
|  | No | No | No | Yes | Yes | No | No | No | No | No | No | No | No |
|  | Yes | Yes | No | Yes | No | Yes | No | Yes | No | Yes | No | Yes | No |
|  | Yes | Yes | No | Yes | No | Yes | No | Yes | No | Yes | No | Yes | No |
|  | Yes | Yes | No | Yes | No | Yes | No | Yes | No | Yes | No | Yes | No |
|  | Yes | Yes | No | Yes | No | Yes | No | Yes | No | Yes | No | Yes | No |
|  | Yes | Yes | No | Yes | No | Yes | No | Yes | No | Yes | No | Yes | No |
|  | Yes | Yes | No | Yes | No | Yes | No | Yes | No | Yes | No | Yes | No |
|  | No | No | No | No | No | No | No | No | No | No | No | No | No |
|  | No | No | No | No | No | No | No | No | No | No | No | No | No |
|  | Yes | Yes | No | Yes | No | Yes | No | Yes | No | Yes | No | Yes | No |
|  | Yes | Yes | No | Yes | No | Yes | No | Yes | No | Yes | No | Yes | No |
|  | Yes | Yes | No | Yes | No | Yes | No | Yes | No | Yes | No | Yes | No |
|  | Yes | Yes | No | Yes | No | Yes | No | Yes | No | Yes | No | Yes | No |
|  | No | No | No | No | No | No | No | No | No | No | No | No | No |
|  | Yes | Yes | No | Yes | No | Yes | No | Yes | No | Yes | No | Yes | No |
|  | No | No | No | No | No | No | No | No | No | No | No | No | No |
|  | No | No | No | No | No | No | No | No | No | No | No | No | No |
|  | Yes | Yes | No | Yes | No | Yes | No | Yes | No | Yes | No | Yes | No |
|  | Yes | Yes | No | Yes | No | Yes | No | Yes | No | Yes | No | Yes | No |
|  | Yes | Yes | No | Yes | No | Yes | No | Yes | No | Yes | No | Yes | No |
|  | Yes | Yes | No | Yes | No | Yes | No | Yes | No | Yes | No | Yes | No |
|  | No | No | No | No | No | No | No | No | No | No | No | No | No |
|  | No | No | No | No | No | No | No | No | No | No | No | No | No |
|  | Yes | Yes | No | Yes | No | Yes | No | Yes | No | Yes | No | Yes | No |
|  | Yes | Yes | No | Yes | No | Yes | No | Yes | No | Yes | No | Yes | No |
|  | Yes | Yes | No | Yes | No | Yes | No | Yes | No | Yes | No | Yes | No |
|  | No | No | No | No | No | No | No | No | No | No | No | No | No |

**Table S5**: Concordance of reference recommendations generated from the 2018 ESC/ESH guideline after leave-one-out sensitivity analysis

| **Code** | **Concordance of recommendation** | **Concordance of recommendation after exclusion of ‘MAL’** | **Does excluding 'MAL' make a difference in concordance of recommendation?** | **Concordance of recommendation after exclusion of ‘BRN’** | **Does excluding 'BRN' make a difference in concordance of recommendation?** | **Concordance of recommendation after exclusion of ‘SGP’** | **Does excluding 'SGP' make a difference in concordance of recommendation?** | **Concordance of recommendation after exclusion of ‘THA’** | **Does excluding 'THA' make a difference in concordance of recommendation?** | **Concordance of recommendation after exclusion of ‘IDN’** | **Does excluding 'IDN' make a difference in concordance of recommendation?** | **Concordance of recommendation after exclusion of ‘VNM’** | **Does excluding 'VNM' make a difference in concordance of recommendation?** |
| --- | --- | --- | --- | --- | --- | --- | --- | --- | --- | --- | --- | --- | --- |
|  | Yes | Yes | No | Yes | No | Yes | No | Yes | No | Yes | No | Yes | No |
|  | Yes | Yes | No | Yes | No | Yes | No | Yes | No | Yes | No | Yes | No |
|  | Yes | Yes | No | Yes | No | Yes | No | Yes | No | Yes | No | Yes | No |
|  | No | No | No | No | No | No | No | Yes | Yes | Yes | Yes | Yes | Yes |
|  | Yes | Yes | No | Yes | No | Yes | No | Yes | No | Yes | No | Yes | No |
|  | No | No | No | No | No | No | No | No | No | No | No | No | No |
|  | No | No | No | No | No | No | No | No | No | Yes | Yes | No | No |
|  | No | No | No | No | No | No | No | No | No | Yes | Yes | No | No |
|  | Yes | Yes | No | Yes | No | Yes | No | Yes | No | Yes | No | Yes | No |
|  | Yes | Yes | No | Yes | No | Yes | No | Yes | No | Yes | No | Yes | No |
|  | No | No | No | No | No | No | No | No | No | No | No | No | No |
|  | No | No | No | No | No | No | No | No | No | No | No | No | No |
|  | No | No | No | No | No | No | No | No | No | No | No | No | No |
|  | Yes | Yes | No | Yes | No | Yes | No | Yes | No | Yes | No | Yes | No |
|  | Yes | Yes | No | Yes | No | Yes | No | Yes | No | Yes | No | Yes | No |
|  | Yes | Yes | No | Yes | No | Yes | No | Yes | No | Yes | No | Yes | No |
|  | Yes | Yes | No | Yes | No | Yes | No | Yes | No | Yes | No | Yes | No |
|  | Yes | Yes | No | Yes | No | Yes | No | Yes | No | Yes | No | Yes | No |
|  | Yes | Yes | No | Yes | No | Yes | No | Yes | No | Yes | No | Yes | No |
|  | Yes | Yes | No | Yes | No | Yes | No | Yes | No | Yes | No | Yes | No |
|  | Yes | Yes | No | Yes | No | Yes | No | Yes | No | Yes | No | Yes | No |
|  | No | No | No | No | No | No | No | No | No | No | No | No | No |
|  | No | No | No | No | No | No | No | No | No | No | No | No | No |
|  | No | No | No | No | No | No | No | No | No | No | No | No | No |
|  | No | No | No | No | No | No | No | No | No | No | No | No | No |
|  | No | No | No | No | No | No | No | No | No | No | No | No | No |
|  | No | No | No | No | No | No | No | No | No | No | No | No | No |
|  | No | No | No | No | No | No | No | No | No | No | No | No | No |
|  | No | No | No | Yes | Yes | No | No | No | No | No | No | No | No |
|  | No | No | No | Yes | Yes | No | No | No | No | No | No | No | No |
|  | Yes | Yes | No | Yes | No | Yes | No | Yes | No | Yes | No | Yes | No |
|  | Yes | Yes | No | Yes | No | Yes | No | Yes | No | Yes | No | Yes | No |
|  | Yes | Yes | No | Yes | No | Yes | No | Yes | No | Yes | No | Yes | No |
|  | Yes | Yes | No | Yes | No | Yes | No | Yes | No | Yes | No | Yes | No |
|  | Yes | Yes | No | Yes | No | Yes | No | Yes | No | Yes | No | Yes | No |
|  | Yes | Yes | No | Yes | No | Yes | No | Yes | No | Yes | No | Yes | No |
|  | No | No | No | No | No | No | No | No | No | No | No | No | No |
|  | No | No | No | No | No | No | No | No | No | No | No | No | No |
|  | Yes | Yes | No | Yes | No | Yes | No | Yes | No | Yes | No | Yes | No |
|  | Yes | Yes | No | Yes | No | Yes | No | Yes | No | Yes | No | Yes | No |
|  | Yes | Yes | No | Yes | No | Yes | No | Yes | No | Yes | No | Yes | No |
|  | Yes | Yes | No | Yes | No | Yes | No | Yes | No | Yes | No | Yes | No |
|  | Yes | Yes | No | Yes | No | Yes | No | Yes | No | Yes | No | Yes | No |
|  | Yes | Yes | No | Yes | No | Yes | No | Yes | No | Yes | No | Yes | No |
|  | Yes | Yes | No | Yes | No | Yes | No | Yes | No | Yes | No | Yes | No |
|  | Yes | Yes | No | Yes | No | Yes | No | Yes | No | Yes | No | Yes | No |
|  | No | No | No | No | No | No | No | No | No | No | No | No | No |
|  | No | No | No | No | No | No | No | No | No | No | No | No | No |
|  | Yes | Yes | No | Yes | No | Yes | No | Yes | No | Yes | No | Yes | No |
|  | Yes | Yes | No | Yes | No | Yes | No | Yes | No | Yes | No | Yes | No |
|  | Yes | Yes | No | Yes | No | Yes | No | Yes | No | Yes | No | Yes | No |
